# Tissue tropism and functional adaptation of the SARS-CoV-2 spike protein in a fatal case of COVID-19

**DOI:** 10.1128/jvi.00857-25

**Published:** 2025-10-31

**Authors:** Katherine E. E. Johnson, Sydney Stein, Rita Afriyie Boateng, Shilpi Jain, Sabrina C. Ramelli, Trevor Stantliff, Shelly Curran, Marcos J. Ramos-Benítez, Andrew P. Platt, Stephanie Banakis, Wei Wang, Stephen M. Hewitt, Christa Zerbe, Steven M. Holland, Elizabeth M. Kang, Manmeet Singh, Emmie de Wit, William A. Lauer, Eric C. Rouchka, Melissa Smith, Mehul S. Suthar, Daniel S. Chertow, Elodie Ghedin

**Affiliations:** 1 Systems Genomics Section, Laboratory of Parasitic Diseases, Division of Intramural Research, National Institute of Allergy and Infectious Diseases, National Institutes of Health 2511, Bethesda, Maryland, USA; 2 Critical Care Medicine Department, Emerging Pathogens Section, Clinical Center, National Institutes of Health 2511, Bethesda, Maryland, USA; 3 Laboratory of Virology, Division of Intramural Research, National Institute of Allergy and Infectious Diseases, National Institutes of Health 2511, Hamilton, Montana, USA; 4 Division of Infectious Diseases, Department of Pediatrics, Center for Childhood Infections and Vaccines, Children’s Healthcare of Atlanta, Emory University School of Medicine 12239, Atlanta, Georgia, USA; 5 Emory Vaccine Center, Emory University 1371, Atlanta, Georgia, USA; 6 Emory National Primate Research Center 145235, Atlanta, Georgia, USA; 7 Laboratory of Pathology, Center for Cancer Research, National Cancer Institute, National Institutes of Health 2511, Bethesda, Maryland, USA; 8 Division of Intramural Research, Laboratory of Clinical Immunology and Microbiology, National Institute of Allergy and Infectious Diseases, National Institutes of Health 2511, Bethesda, Maryland, USA; 9 Department of Biochemistry and Molecular Genetics, University of Louisville 5170, Louisville, Kentucky, USA; 10 Department of Microbiology and Immunology, Emory University 1371, Atlanta, Georgia, USA; St Jude Children's Research Hospital, Memphis, Tennessee, USA

**Keywords:** viral evolution, viral intrahost diversity, tissue tropism, coronavirus

## Abstract

Systemic spread of severe acute respiratory syndrome coronavirus 2 (SARS-CoV-2) to extrapulmonary tissues has been observed following acute infections. Autopsy studies further indicate tissue-specific virus diversity, including in immune-privileged sites. Questions remain on the viral dynamics leading to the tissue tropism of SARS-CoV-2, including evolutionary trajectories and functional adaptations that could impact persistence and transmission. In this study, we characterized SARS-CoV-2 genomes from 27 distinct tissues collected from an autopsy case where the patient had a primary immune deficiency. We identified tissue-specific virus genotypes, in some instances coexisting within the same sites, with mutations primarily in the receptor-binding domain of the spike protein. Protein simulations and isolation of infectious virus indicate combinations of spike substitutions that would lead to increased protein stability and stronger binding of the virus to host cells. This highlights the importance of studying patients with weakened immune responses where potential tissue reservoirs provide an environment permissive for SARS-CoV-2 evolution and diversification.

**IMPORTANCE:**

Persistent severe acute respiratory syndrome coronavirus 2 (SARS-CoV-2) infections in immunocompromised individuals are considered a potential source of new viral variants. Beyond the respiratory tract, the virus can spread within days to organs like the brain, heart, and kidneys, where distinct tissue microenvironments may further drive viral evolution and the emergence of new mutations. In this study, we compared the genetic diversity of SARS-CoV-2 genomic RNA isolated from 27 distinct tissue sites collected from an individual with a weakened immune system. By linking viral population dynamics across these tissue sites, we defined the extent of compartmentalization during multi-organ spread, highlighting how non-respiratory tissues can impact SARS-CoV-2 diversification.

## INTRODUCTION

Severe acute respiratory syndrome coronavirus 2 (SARS-CoV-2) primarily infects the respiratory epithelium. However, SARS-CoV-2 is not confined to the respiratory tract and has been detected in various tissues, including ocular, cardiac, and neuronal compartments (1–4). These tissues express different levels of SARS-CoV-2′s primary receptor, angiotensin-converting enzyme 2 (ACE2), and its activating protease, transmembrane serine protease 2 (TMPRSS2), which are important for viral entry into cells (5, 6). The availability of the ACE2 and TMPRSS2 on the surface of the cells, along with the spatial structure of tissues, whether the tissue is considered immune privileged, and the expression of intracellular cofactors can all shape the types of SARS-CoV-2 variants that enter, replicate, and circulate within a host.

The mechanisms of SARS-CoV-2 transmission to non-respiratory tissues remain unclear, although neuronal, hematogenous, and cellular trafficking transmission routes have all been proposed (7–10). SARS-CoV-2 can spread to extrapulmonary tissues within days of symptom onset in both healthy individuals and those with comorbidities (1). The presence of SARS-CoV-2 in extrapulmonary tissues following acute infections has led to questions on whether systemic spread of the virus is linked to the extensive set of symptoms associated with post-acute sequelae of COVID-19, also known as “long-COVID,” where individuals report symptoms for weeks to months following infection (11). Typically, infectious SARS-CoV-2 particles are cleared within 9 days, but viral RNA can be detected in the gastrointestinal tract for weeks and sometimes months following infection (12, 13). Furthermore, autopsy studies have found that SARS-CoV-2 can also persist in immune-privileged tissues such as the brain (1) and generate tissue-specific diversity, where unique genotypes are observed in the different tissue sites (1–3), leading to the possibility that non-respiratory tissues may act as reservoirs, allowing SARS-CoV-2 to persist at nearly undetectable levels within individuals.

Persistent SARS-CoV-2 infections may be more common than initially predicted, are associated with a higher risk of long-term symptoms, and have important implications for the evolution and spread of the virus (14, 15). Understanding the tissue tropism of SARS-CoV-2 is crucial for characterizing its evolutionary trajectories, persistence, transmission, and disease severity. In this study, we obtained whole-genome sequences of SARS-CoV-2 isolated from 27 distinct tissues collected from an autopsy case. We characterized viral dynamics across the different tissue compartments, including variant richness, evolutionary changes, and shared virus diversity. Additionally, we assessed the functional effects of spike (S) mutations that accumulate during infection using protein simulations and binding and internalization assays with infectious isolates.

## RESULTS

### Multiple SARS-CoV-2 genotypes are identified across tissues in an autopsy case

On 29 September 2021, a 57-year-old male patient presented to a Florida hospital with fever, lethargy, shortness of breath, and cough. His prior medical history was significant for daily immunosuppression with sirolimus following a matched unrelated donor hematopoietic stem cell transplant (HSCT) in January 2021 to treat chronic granulomatous disease (CGD), a primary immune deficiency, with associated recurrent respiratory infections, severe bronchiectasis, and respiratory insufficiency requiring home oxygen and noninvasive mechanical ventilation. Upon presentation to the emergency room, he tested positive for SARS-CoV-2 7 days following his first COVID-19 vaccine and was admitted for monoclonal antibody and remdesivir therapy. On 4 October, he was discharged following resolution of his acute symptoms. During 21−27 October, he was readmitted with recurrent respiratory symptoms, a new right upper lobe infiltrate, and a negative SARS-CoV-2 test. He was treated with intravenous antibiotics and prednisone 40 mg orally twice daily. Following hospital discharge, the patient moved to New York to be closer to family. On 23 November, he was admitted to a New York hospital with worsening shortness of breath and hypoxia. He again tested positive for SARS-CoV-2. The patient died on 28 November from respiratory failure and on 2 December underwent autopsy at the National Institutes of Health Clinical Center following consent from next of kin.

At autopsy, 73 distinct tissue sites were collected. Of these, 61 had SARS-CoV-2 RNA copies above the detection limit (see Table S1). We selected 32 sites for additional analyses where SARS-CoV-2 genome copies ranged from 1.38 to 5.21 log_10_ nucleocapsid (N) gene copies per nanogram of RNA, with an average of 3.89 log_10_ N copies per nanogram of RNA. SARS-CoV-2 subgenomic RNA (sgRNA) was detected in 28 of 32 samples, indicating active viral replication occurred in these tissues (Table S1). Nucleocapsid gene copies and sgRNA were positively correlated (*r* = 0.99, *P* < 2.2e − 16, Pearson) (see Fig. S1A).

Droplet digital PCR (ddPCR) results for each tissue site indicated viral loads similar to those of an acute infection (≤14 days) (1). To determine whether the patient died with a persistent SARS-CoV-2 infection lasting since the initial infection in late September 2021, or an acute reinfection with SARS-CoV-2, we performed phylogenetic analyses using whole-genome sequencing data of SARS-CoV-2 RNA isolated from the 32 distinct tissue sites (Table S1). All 32 samples were sequenced twice using ARTIC V4 primers and the Illumina sequencing platform, and all, except for the tongue and pericardium samples, were sequenced once using long-read PacBio HiFi viral sequencing (16). Although the long-read (PacBio) and short-read (Illumina) data had similar mean and median read depths (Fig. S1B and C), the long-read data had better read-depth evenness across the genome (Fig. S1D), which was not significantly correlated with ddPCR results (*r* = −0.16, *P*-value = 0.4, Pearson) (Fig. S1E), indicating that the long-read data were better at capturing low-abundance viral genomes. Therefore, we used the long-read data for variant analyses and confirmed our findings with the short-read data when read coverage was sufficient.

Five samples, including the basilar artery, spleen, dura mater, cervical spinal cord, and thoracic aorta, only had 10× read depths across 10.3%, 35.5%, 43.2%, 58.8%, and 59.4% of the genome, respectively, and were therefore not used for any genomic analyses. The 27 of 32 remaining samples had ≥10× read depths across ≥80% of the genome and were used for consensus and phylogenetic analyses.

**Fig 1:**
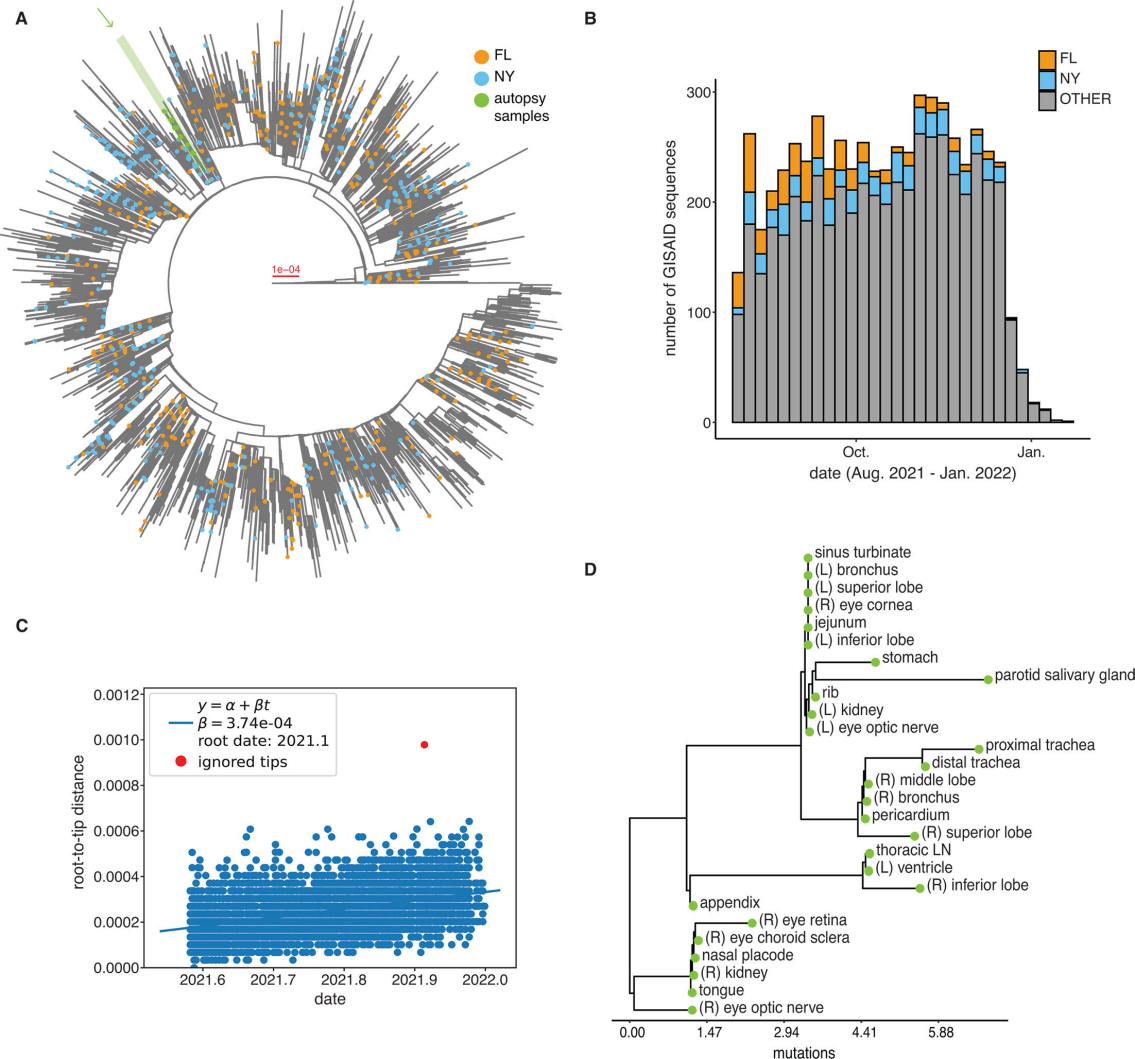
Multiple SARS-CoV-2 genotypes are identified across tissues in an autopsy case. (**A**) Maximum-likelihood phylogenetic tree of 27 consensus sequences from the autopsy case and 6,000 randomly selected AY.119 consensus sequences circulating in the USA between 1 August 2021 and 31 January 2022. Color indicates if the sample was collected in Florida (FL, orange), New York (NY, blue), a state outside of NY or FL (no point), or our autopsy case (green). Outlier sequences are excluded from the tree. (**B**) The proportion of 6,000 randomly sampled AY.119 sequences from GISAID collected in the USA from 1 August 2021 to 31 January 2022. Color indicates if the sample was collected and sequenced in New York (NY, purple), Florida (FL, orange), or a different state (gray). (**C**) The TreeTime output of the root-to-tip distance versus the collection date. Outliers and autopsy samples were ignored (red points). (**D**) Maximum-likelihood phylogenetic tree of the SARS-CoV-2 virus consensus sequences collected and sequenced from 27 different tissue sites. The *x*-axis represents the estimated number of substitutions from the shared ancestral node.

At the time of the patient’s death, Omicron BA.1 lineages were beginning to displace Delta lineages. Using NextClade, we found that all 27 samples were positive for an AY.119 (Delta, 21J) lineage infection. To determine the location (and general timing) of the infection, we generated a maximum-likelihood phylogenetic tree using IQ-TREE with the 27 SARS-CoV-2 consensus sequences collected from 27 different tissue sites and 6,000 randomly selected, complete AY.119 consensus sequences circulating from 1 August 2021 to 31 January 2022 in the USA (Fig. 1A and B). Root-to-tip distances of the AY.119 lineages increased over time, with an estimated substitution rate of 3.76e − 04 substitutions/site/year (Fig. 1C).

The closest sequences were collected from Pennsylvania, Texas, Virginia, and Ohio. Therefore, we were unable to determine the location of infection from the phylogenetic analyses, which limited our ability to infer the length of infection. Samples from the patient were monophyletic but differed in the number of mutations from the shared ancestor, with the estimated number of nucleotide substitutions ranging from 1.19 to 6.83 from the root (Fig. 1D), indicating the coexistence of multiple virus genotypes circulating within the host across the 27 tissues at a single point in time.

### Consensus diversity is primarily in the spike coding region

**Fig 2:**
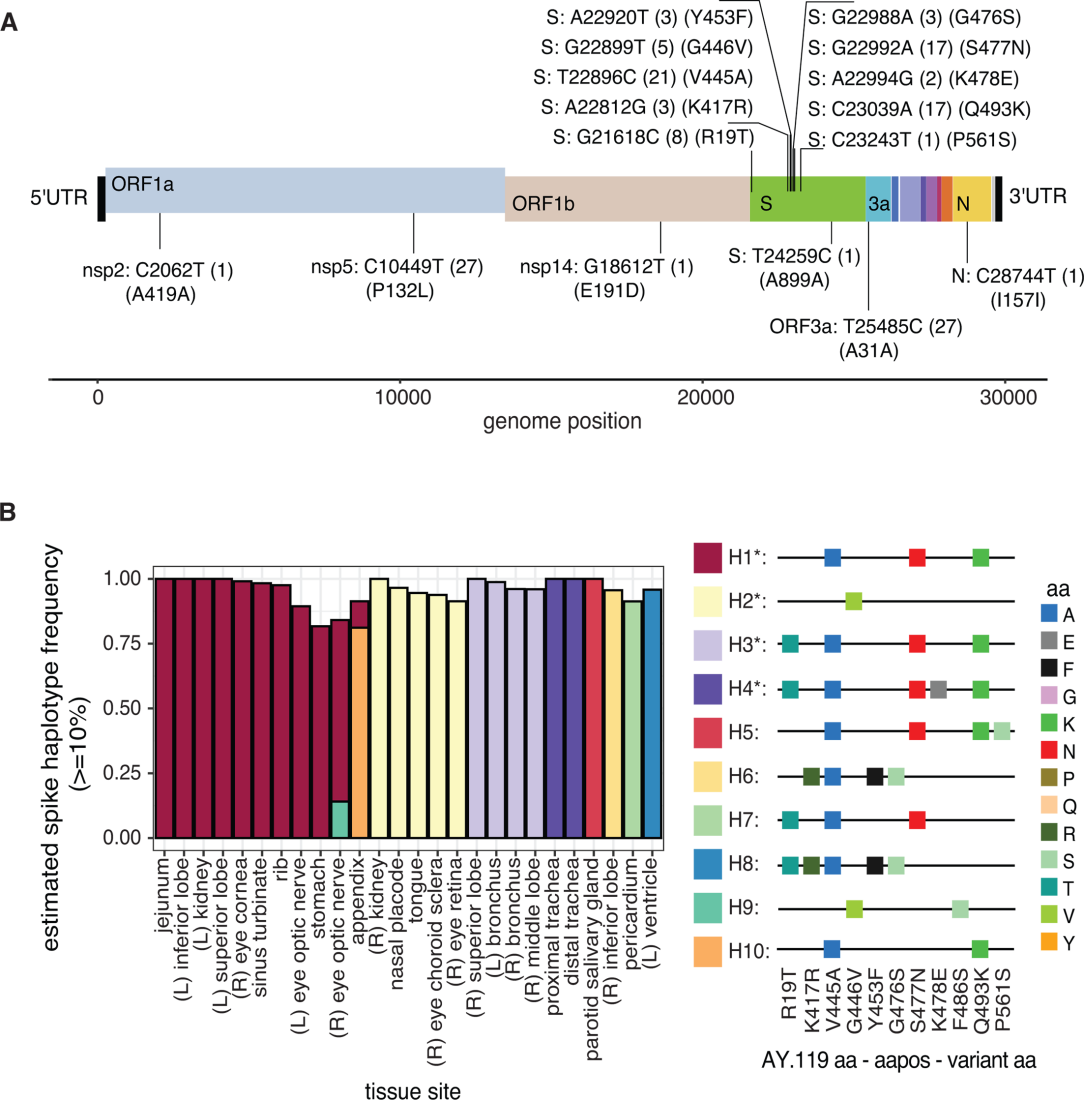
Consensus diversity is primarily in the spike coding region. (**A**) Consensus mutations along the SARS-CoV-2 genome. Mutations are determined by comparing each nucleotide position to the AY.119 consensus reference (Materials and Methods). Each mutation is labeled with the coding region: AY.119 nucleotide–nucleotide position–variant nucleotide. The number of samples with the variant and the amino acid (aa) information (AY.119 aa–aa position–variant aa) is provided in parentheses. Both synonymous and nonsynonymous substitutions are labeled. Open reading frames and coding regions are represented by different colored boxes; only regions with variants are labeled. UTR, untranslated region; ORF, open reading frame; 3a, open reading frame 3a (ORF3a). (**B**) The estimated spike haplotype (H) frequency (*y*-axis) calculated using CliqueSNV for each virus sequenced from each tissue site (*x*-axis). Only spike haplotypes predicted to be present at ≥10% are provided. Color indicates the spike haplotype (H1–H10) identified in each tissue site, with the amino acid residues for each haplotype outlined to the right. Only nonsynonymous amino acid substitutions are provided. Asterisks (*) mark haplotypes found in multiple samples. See also Table 1 and Fig. S2 and S3.

Across the 27 tissue sites, we identified all SARS-CoV-2 nucleotide positions with a consensus change compared to the AY.119 reference (Fig. 2A). Two mutations were strain-specific (nsp5: C10449T [P132L] and ORF3a: T25485C) and were found to be fixed (>98%) across all 27 tissue sites (see Fig. S2A). Five mutations (nsp2: C2062T [A419A], nsp14: G18612T [E191D], S: C23243T [P561S], S: T24259C [A899A], and N: C28744T [I157I]) reached ≥50% in only one sample. The remaining nine mutations varied in the number of samples in which they were found as a consensus change and were all nonsynonymous mutations in the spike coding region. One substitution (R19T) is in the N-terminal domain (NTD), and eight substitutions (K417R, V445A, G446V, Y453F, G476S, S477N, K478E, and Q493K) are in the receptor-binding domain (RBD) of spike (Fig. 2A). We confirmed that all samples had sufficient coverage to determine the major nucleotide at each variant position, ensuring the differences in the appearance of a consensus mutation are not due to insufficient read depths in the spike region (Fig. S2A). Several consensus variants were minor variants in other tissue sites (Fig. S2A). Interestingly, the presence of specific substitutions was dependent on the tissue site. For example, G446V and A419A (synonymous change) were not identified in lung, tracheal, or cardiac samples above our limits of detection. In contrast, K417S, G476S, K478E, and A899A (synonymous change) were only found in the lung, tracheal, cardiac, and gastrointestinal tissues (Fig. S2A).

To determine if spike mutations were linked, we assembled predicted SARS-CoV-2 spike haplotypes (H) circulating in the tissue sites at frequencies of at least 10% using CliqueSNV (Materials and Methods) (17). Ten unique spike haplotypes (10%–100%) were identified across the tissue sites with different combinations of 11 nonsynonymous amino acid substitutions in the spike (Table 1, Fig. 2B). Four haplotypes (H1–H4) were found in at least two samples: H1 (V445A-S477N-Q493K) in the jejunum, sinus turbinate, rib, stomach, appendix, left optic nerve, inferior lung lobe, kidney, and superior lung lobe, and the right cornea and optic nerve; H2 (G446V) in the right kidney, nasal placode, tongue, right choroid sclera, and right retina; H3 (R19T-V445A-S477N-Q493K) in the right superior lung lobe, left and right bronchus, and right middle lung lobe; and H4 (R19T-V445A-S477N-K478E-Q493K) in the proximal and distal trachea. Four substitutions (R19T, V445A, S477N, and Q493K) were found in at least four distinct haplotypes in different combinations and with other spike mutations (Fig. 2B).

**TABLE 1:** Spike haplotypes identified

Haplotype ID	Spike mutation(s)	Tissue site(s)
H1	V445A-S477N-Q493K	Jejunum, sinus turbinate, rib, stomach, appendix, (L) optic nerve, inferior lobe, kidney, superior lobe, (R) cornea, (R) optic nerve
H2	G446V	(R) kidney, nasal placode, tongue, (R) choroid sclera, (R) retina
H3	R19T-V445A-S477N-Q493K	(R) superior lobe, (L) and (R) bronchus, (R) middle lobe
H4	R19T-V445A-S477N-K478E-Q493K	Proximal and distal trachea
H5	V445A-S477N-Q493K-P561S	Parotid salivary gland
H6	K417R-V445A-Y453F-G476S	(R) inferior lobe
H7	R19T-V445A-S477N	Pericardium
H8	R19T-K417R-V445A-Y453F-G476S	(L) ventricle
H9	G446V-F486S	(R) eye optic nerve
H10	V445A-Q493K	Appendix

The 11 spike substitutions identified in the patient samples were rarely found in other SARS-CoV-2 sequences circulating between January 2021 and January 2024 (cov-spectrum.org). Of these, only 477N (BA.1, BA.2, BA.4, BA.5, XBB.1.5) and 486S (BA.2) are classified as lineage-defining mutations of Omicron strains circulating after AY.119 (Fig. S3A). Two substitutions, G446V and Y453F, were identified as emergent mutations in Delta infections following monoclonal antibody treatment and were found to decrease neutralization efficiency (18). Although we do not observe the exact lineage-defining mutations, later SARS-CoV-2 strains harbor different lineage-specific substitutions at residues 19, 417, 445, 446, 478, and 493 in the spike protein (Fig. S3B), emphasizing that the mutations identified in our autopsy samples occur at mutational hotspots within the spike coding sequence.

### Most combinations of spike substitutions are associated with increased protein stability and higher binding energy to the ACE2 receptor

**Fig 3:**
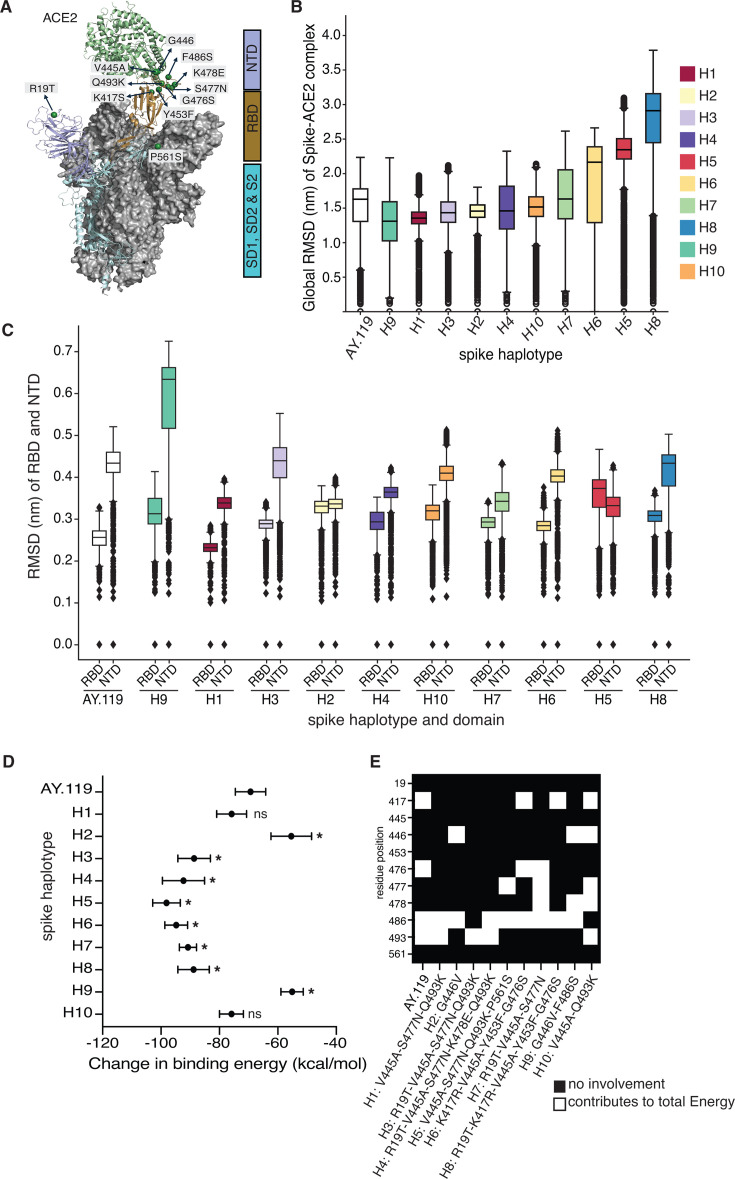
Most combinations of spike substitutions are associated with increased protein stability and higher binding energy to the ACE2 receptor. (**A**) Structural mapping of individual spike substitutions on the 3D AY.119 spike-ACE2 complex. Substitutions are represented as the AY.119 amino acid, amino acid position, and variant amino acid. The spike domains are represented by color. NTD, N-terminal domain (periwinkle); RBD, receptor-binding domain (brown); SD1, sub-domain 1 (blue); and SD2, sub-domain 2 (blue). (**B, C**) Distributions of averaged root mean square deviations (RMSD) (*y*-axis) for the (**B**) complete spike-ACE2 complex or the (**C**) NTD and RBD of the spike across all spike sequences (*x*-axis). Average RMSD values were calculated for 100 individual frames at every nanosecond of an 80 ns molecular dynamics simulation time (*n* = 8,000 per spike haplotype). Global RMSD values (**B**) for all mutant haplotypes were significantly different from the AY.119 reference (adj. *P*-value <0.0001, Kruskal-Wallis). Color indicates the haplotype and matches the haplotype colors outlined in Fig. 2. All boxplots represent the median (middle line), first and third quartiles (box), 1.5 * interquartile range (whiskers), and outliers (points). (**D**) The mean and standard deviation of the change in binding free energy (kcal/mol) (*x*-axis) between the entire spike structure and ACE2 for each spike sequence (*y*-axis) using molecular mechanics Poisson-Boltzmann surface area. Non-significant differences between AY.119 and each mutant spike haplotype are noted with ns, while significant differences are marked with * and indicate a *P*-value <0.0001 using a one-way ANOVA test. (**E**) A heatmap of the decomposed energetic interactions for each residue (*y*-axis) where a consensus variant was found in our data set. AY.119 reference residues include R19, K417, V445, G446, Y453, L455, G476, S477, K478, Q493, and P561. Only variant residues are outlined for each mutant spike haplotype along the *x*-axis. If not provided, the residue is the reference AY.119 residue. White boxes indicate the residue is significantly involved in the total binding energy. See also Fig. S4.

Considering that most of the substitutions identified were in the receptor-binding domain of the spike protein, we performed protein simulations on the SARS-CoV-2 spike and human ACE2 receptor complex to test how the mutant spike haplotypes impact the stability and function of the spike protein. We first remodeled the spike-ACE2 complex structure (Protein Data Bank [PDB] ID: 7W98) to fill in missing residues. Eight of the 11 substitutions clustered on unstructured loops of the RBD, a region that directly interacts with ACE2 (Fig. 3A). The remaining two were buried in the loops of the NTD (R19T) and sub-domain 1 (SD1) (P561S) (Fig. 3A).

The structural stability of the spike-ACE2 complex for the AY.119 reference and 10 mutant haplotypes (Table 1) was estimated using the global (whole S-ACE2 complex) and domain-based root mean square deviation (RMSD) analysis, which designates the atomic position fluctuations (Fig. 3B and C; Fig. S4). All systems attained convergence after 10 ns, indicating reliable trajectories. A significant difference in RMSD (adj. *P*-value < 0.0001, Kruskal-Wallis) was established between AY.119 and all mutant haplotypes. Spike sequences for H9 (G446V-F486S, 1.30 nm), H1 (V445A-S477N-Q493K, 1.35 nm), and H3 (R19T-V445A-S477N-Q493K, 1.42 nm) exhibited highly stable or constrained conformations compared to AY.119 (1.51 nm). H4 (R19T-V445A-S477N-K478E-Q493K, 1.49 nm) and H7 (R19T-V445A-S477N, 1.51 nm) showed similar structural dynamics to AY.119. Conversely, H8 (R19T-K417R-V445A-Y453F-G476S, 2.61 nm), H5 (V445A-S477N-Q493K-P561S, 2.24 nm), and H6 (K417R-V445A-Y453F-G476S, 1.88 nm) had the highest structural deviation and potential instability, or increased flexibility compared to AY.119. The higher RMSD is due to the increased flexibility of atoms in the subunit 1 region (S1) of the spike, particularly at sites that do not interact with ACE2 (i.e., NTD) (Fig. 3C; Fig. S4A).

Individual residue fluctuations were analyzed by calculating root mean square fluctuation (RMSF) to investigate the effect of each substitution on the spike-ACE2 residue interactions. Most residues in the SARS-CoV-2 spike RBD exhibit minimal fluctuations, with RMSF less than 2.0 Å (Fig. S4B). However, relatively high RMSFs are shown across the residues 14–24, 69–77, and 142–149 (in the NTD region) and were prominent in H5 and H8 sequences relative to the other sites and the AY.119 reference.

To determine the impact of the spike substitutions on spike-ACE2 interactions, we calculated Gibbs free binding energy differences between the ACE2 region (atoms 17,458–26,974) and the spike (atoms 1–17,457) (Fig. 3D and E). All 10 mutant haplotypes exhibited significant changes in Gibbs free energy (*P*-value <0.0001, one-way ANOVA) with respect to AY.119 (-69.4 kcal mol^−1^), except for H1 (−75.4 kcal mol^−1^) and H10 (−70.6 kcal mol^−1^) (Fig. 3D). In AY.119, residue positions K417, G476, F486, and Q493 contributed significantly to the overall energy profile between spike and ACE2 (Fig. 3E). Notably, residue 417 lost its significance in most haplotypes (H1–H5, H7) (Fig. 3E). Across all haplotypes, including AY.119, residues at 486 and 493 showed the highest contribution to the binding of spike to ACE2, indicating their importance in structural stability (Fig. 3E).

### Combinations of spike substitutions impact the viral binding and internalization of infectious isolates

Infectious virus isolates were plaque-purified in VeroE6-TMPRSS2 cells from 6 of the 16 tissue sites tested (Table S2). Depending on the tissue site, SARS-CoV-2 RNA was isolated and sequenced from one to three purified plaques (Table S3). Isolate 1 spike sequence (R19T-V445A-S477N-K478E-Q493K) was found in 9 of 16 plaque isolates (Table S2) and was the dominant spike haplotype in the sequencing data of the proximal and distal trachea (see H4 genotype in Fig. 2B). The spike sequence of isolate 2 (V445A-S477N-Q493K) was the predominant haplotype in the original sequencing data (H1 genotype in Fig. 2B) but was only found in a sinus turbinate and tongue plaque (plaque #6). The remaining three isolates’ spike sequences had additional substitutions (E406D, L455F, T573I, G1267R) that were not observed in the consensus sequences of the original sequencing data. Although the E406D, L455F, and T573I substitutions did not reach ≥50% in the original sequencing data, they were present as minor variants (Fig. S5), indicating they may have an advantage in cell lines.

**Fig 4:**
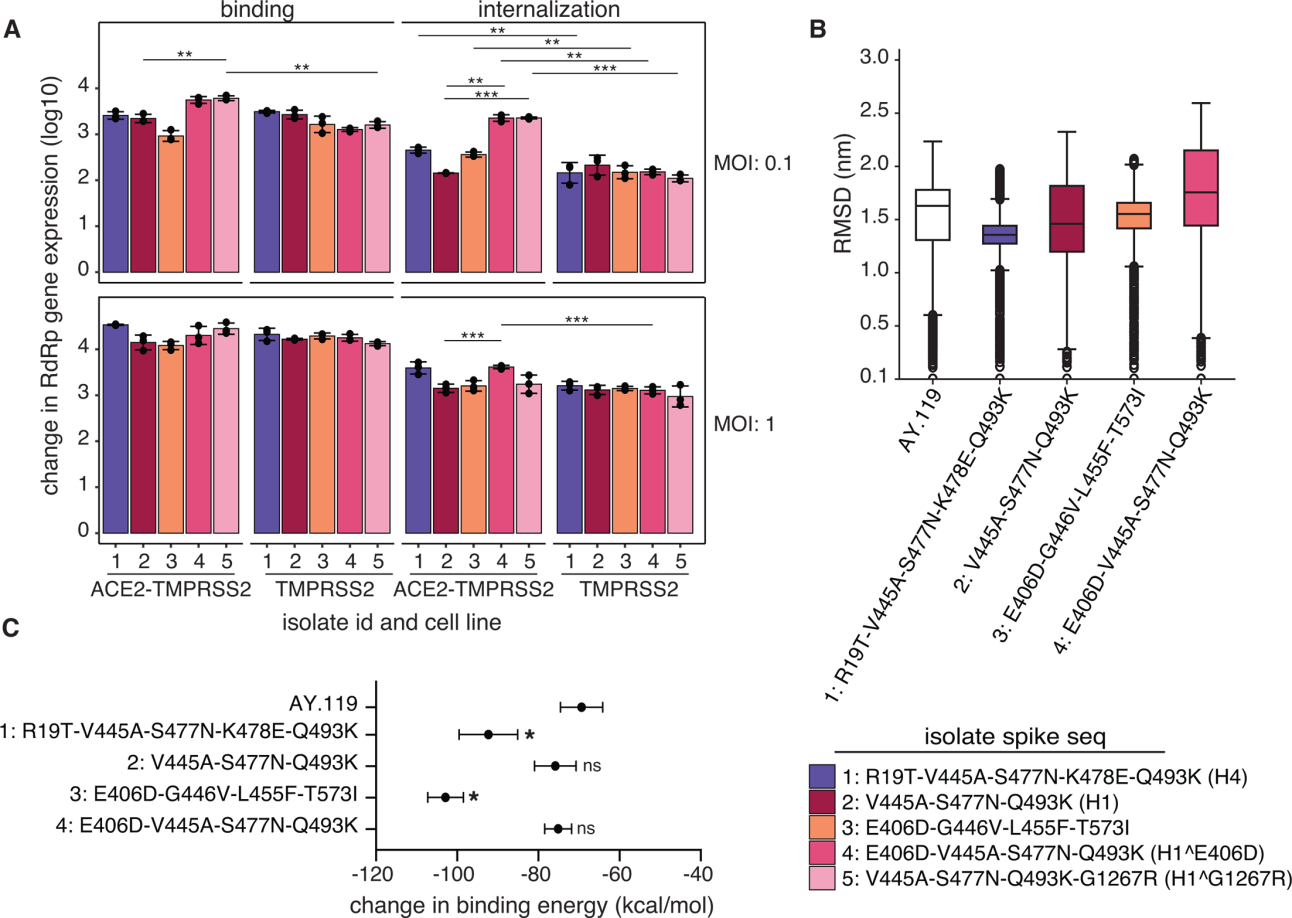
Combinations of spike substitutions impact the binding and internalization of infectious isolates. (**A**) The log_10_ fold change in SARS-CoV-2 RdRp gene expression for each plaque-purified isolate (1–5) with respect to mock infections for binding (left) and internalization (right) assays at an MOI of 0.1 (top) and 1 (bottom) in VeroE6 cell lines constitutively expressing human ACE2-TMPRSS2 or TMPRSS2 only (*n* = 3 for each isolate and assay). Data were normalized using GAPDH gene expression, and changes in RdRp gene expression were calculated and compared to those of the mock controls. *P*-values were calculated by comparing each isolate’s binding and internalization measurements to those of isolate 2 or by comparing measurements of each isolate within ACE2-TMPRSS2 and TMPRSS2 cells. Only *P*-values <0.01 are shown in the figure (**P < 0.01, ***P < 0.001). Color indicates the different infectious plaque-purified isolates. The mutant spike sequence is provided for each isolate and includes the AY.119 amino acid residue, amino acid position, and variant amino acid for each variant site. The most similar haplotype sequence found in Fig. 2 is provided in parentheses next to each isolate sequence. See also Tables S2 and S3. (**B**) Distributions of averaged RMSD (*y*-axis) for the complete spike-ACE2 complex across the spike isolates (1–4 only) and the AY.119 reference (*x*-axis). Average RMSD values were calculated for 100 individual frames at every nanosecond of an 80 ns molecular dynamics simulation time (*n* = 8,000). Boxplots represent the median (middle line), first and third quartiles (box), 1.5 * interquartile range (whiskers), and outliers (points). Non-significant differences between AY.119 and each isolate’s spike haplotype are noted with ns, while significant differences (*P*-value <0.0001, one-way ANOVA test) are marked with *. (**C**) The mean and standard deviation of the change in binding free energy (kcal/mol) (*x*-axis) between the entire spike structure and ACE2 for each spike sequence (*y*-axis) using molecular mechanics Poisson-Boltzmann surface area.

We tested the differences in viral binding and internalization by quantifying SARS-CoV-2 RNA-dependent RNA polymerase (RdRp) gene copies via qPCR for five isolates with distinct spike sequences (Fig. 4A). Isolates 1, 4, and 5 all shared the isolate 2 (V445A-S477N-Q493K) backbone but carried one to two additional substitutions (Table S3). Isolate 3 was unique and only shared the E406D substitution with isolate 4. At a multiplicity of infection (MOI) of 0.1, the cell line minimally affected binding, with isolates 4 and 5 having significantly lower binding in the TMPRSS2 cell line (*P* = 0.013, *P* = 0.0045, respectively), suggesting that all isolates were binding efficiently to the endogenously expressed primate ACE2 on the VeroE6-TMPRSS2 cells. In contrast, viral internalization was significantly reduced for isolates 1, 3, 4, and 5 in the TMPRSS2 cells compared to the ACE2-TMPRSS2 cells, especially under low MOI conditions. This result is consistent with prior studies reporting improved viral entry and recovery of low-titer virus when using VeroE6 cells that constitutively express both human ACE2 and TMPRSS2 (19).

Across the isolates, the cell line and MOI influenced the binding and internalization, with only minimal differences observed between the isolates in the VeroE6-TMPRSS2 cells at both an MOI of 0.1 and 1 (Fig. 4A). Given that isolate 2 was the predominant spike sequence in our sequencing data and had three substitutions found in isolates 1, 4, and 5, we compared changes in viral binding and internalization relative to isolate 2. Compared to isolate 2, isolates 4 and 5 had the largest increases in RdRp gene expression at an MOI of 0.1 for both binding (*P* = 0.01 and *P* = 0.003, respectively) and internalization (*P* = 0.0094 and *P* = 0.00084) assays, indicating efficient binding and internalization even at low MOIs. Isolates 4 and 5 each carry one additional substitution from the isolate 2 backbone (V445A-S477N-Q493K), with the addition of E406D and G1267R, respectively. The addition of E406D in isolate 4, located in the receptor-binding domain of the spike, led to an increase in the structural and conformational flexibility compared to the AY.119 reference and the isolate 2 spike sequence (Fig. 4B) but did not decrease the estimated change in binding free energy (Fig. 4C). Interestingly, the E406D substitution also emerged in Delta infections following monoclonal antibody treatment and is associated with reduced neutralization by certain monoclonal antibody therapies (18). These data indicate that the change in structural stability caused by E406D may be beneficial for the binding and entry of the virus in the VeroE6 cells expressing human ACE2 and TMPRSS2. Because G1267R is out of range for the protein complex, the spike sequence from isolate 5 could not be simulated. Isolate 3, which also had the E406D substitution but lacked the isolate 2 backbone, also significantly deviated from the AY.119 reference structure (Fig. 4B); however, it had the lowest binding efficiency in the ACE2-TMPRSS2 cells, especially at a low MOI (Fig. 4A). Together, these results further highlight the coexistence of multiple genotypes circulating within the same tissue and how these mutational differences impact the spike’s structural stability and function, which is influenced by cell line propagation and multiplicity of infection.

### Tissue compartmentalization and mixing of minor variant populations

**Fig 5:**
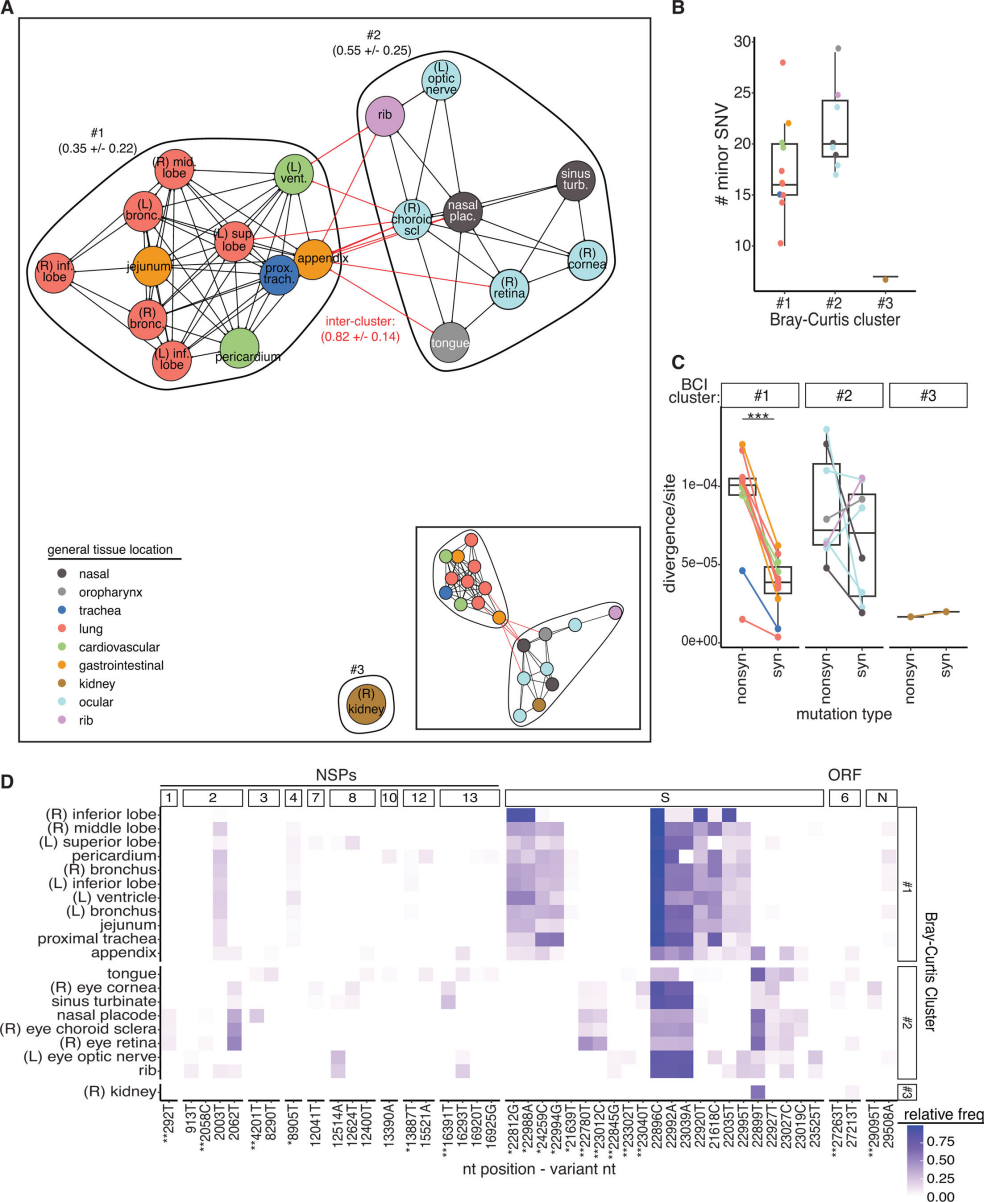
Tissue compartmentalization and mixing of minor variant populations. (**A**) A network visualizing the hierarchical clustering of pairwise BCI values. Links represent a BCI less than the mean BCI for the data set (0.66). Red lines represent connections that span clusters, and black lines indicate connections within a cluster. Each node is colored by the general tissue location. Each cluster is numbered (1–3), and the mean and standard deviation of the intra- and inter-cluster comparisons are provided. BCI values assume independence between variants and were calculated using positions where a variant was present as a minor variant in at least one sample. (**B**) Boxplots of the number of minor (≥5%–49.99%) SNVs (*y*-axis) for the Bray-Curtis clusters. Each point represents a single tissue site. Color represents the general tissue location and is maintained for the figure. (**C**) The nonsynonymous (nonsyn) and synonymous (syn) minor variant divergence for each nucleotide site across the genome, normalized by the total number of available nonsynonymous and synonymous sites, and excluding the untranslated and non-coding regions of the genome. Data are by Bray-Curtis cluster. Each line connects tissue site values. Asterisks represent significance (*P*-values*:* ***<0.001) between noted comparisons using the Mann-Whitney U-test. (**D**) The nucleotide variants found in more than two tissue sites that define or are shared by the three Bray-Curtis clusters. Fill indicates the relative frequency of the given variant (*x*-axis) within each tissue site (*y*-axis). Variants along the *x*-axis are ordered by their cluster association and the total number of samples in which they were identified. Asterisks indicate whether a mutation is unique to cluster 1 (*) or cluster 2 (**). NSP, non-structural protein; ORF, open reading frame. See also Fig. S6.

Given that several consensus-level mutations were found as minor variants in other samples (Fig. S2A), we calculated pairwise Bray-Curtis dissimilarity indices (BCI) using the single-nucleotide variant (SNV) information to quantify the minor variant diversity that is shared across tissue sites (Fig. S6A). The pairwise BCI scores were hierarchically clustered, which separated the tissue sites into two broad general anatomical locations, with samples from cluster 1 (*n* = 11) (lung, tracheal, cardiac, and gastrointestinal tissues) extracted from the thoracic and abdominal regions, while cluster 2 (*n* = 8) was made up of sites mainly from the head, including ocular, nasal, oropharynx regions, and the rib sample (Fig. 5A). Both bone structures extracted (rib and sinus turbinate) fell into cluster 2. The right kidney sample did not cluster with either group, but clustered with samples in cluster 2 when reducing the stringency of SNV cutoffs to 1% and 50×, as most sites fell slightly below the 100× cutoff (Fig. 5A, inset; Fig. S6B). Interestingly, tissues within each cluster often had different consensus sequences (Fig. 1B, Fig. 2A), indicating that even with a high amount of intra-cluster mixing of minor variants, what becomes dominant is dependent on the tissue site. Cluster 1 tissues had lower BCI scores (mean and SD: 0.35 ± 0.22), and more sharing of variants, compared to sites in cluster 2 (mean and SD: 0.55 ± 0.25).

We investigated the differences in minor variant characteristics between tissue sites within each Bray-Curtis cluster (Fig. 5A). Our analysis revealed that all tissue sites varied in the number of minor SNVs, with the tongue (*n* = 29), left superior lobe (*n* = 28), rib (*n* = 25), left optic nerve (*n* = 24), and appendix (*n* = 22) each having more than 20 minor SNVs. Tissue sites from cluster 1 exhibited a lower mean (17.5 ± 4.8) and median (16) minor SNV richness compared to cluster 2 samples (mean: 21.5 ± 4.1, median: 20), although this difference was not statistically significant (*P*-value = 0.056, Mann-Whitney U-test, Fig. 5B). Importantly, minor SNV richness was not significantly correlated with genome copies (*r* = 0.046, *P*-value = 0.85, Pearson, Fig. S6C).

Cluster 1 had significantly higher nonsynonymous divergence than synonymous divergence (*P*-value = 0.00077, Mann-Whitney U-test). In contrast, the left optic nerve, rib, and tongue, which had high SNV richness, exhibited higher synonymous divergence than nonsynonymous divergence. The clusters were primarily defined by the presence and frequency of SNVs in the spike coding region (Fig. 5D). While cluster 2 had more unique mutations, these were mainly shared by only two samples, with no SNVs identified in all eight samples in cluster 2. Conversely, SNVs such as S: 22812G, S: 22988A, and S: 24259C were found in all 11 samples of cluster 1 and none of the samples from clusters 2 or 3. This indicates that cluster 1, despite having fewer unique minor SNVs, had mutations that were more consistently shared across its samples, leading to the lower Bray-Curtis index values. Together, these findings suggest that selection pressures differ significantly across tissues.

## DISCUSSION

We characterized the intrahost genetic diversity of SARS-CoV-2 from distinct tissue sites collected from an autopsy case patient with primary immunosuppression. Phylogenetic analyses of 27 assembled SARS-CoV-2 consensus sequences revealed multiple genotypes circulating across the tissues. The sequences are monophyletic, suggesting divergence within the host rather than coinfection with multiple SARS-CoV-2 genomes. Single-nucleotide accumulation estimates ranged from 1.19 to 6.83 substitutions from the shared ancestral node. This range of estimates fits with the estimated intrahost evolutionary rate of ~35.55 (95% CI: 31.56–39.54) substitutions per year (20), indicating that the infection may have persisted since the initial positive COVID-19 test nearly 2 months prior to death.

Although we cannot determine the exact timing of virus dissemination to non-respiratory tissues, N copy numbers in the respiratory and non-respiratory tissues are similar to those observed in other autopsy cases with acute infections (<14 days) (1). The high copy numbers and sgRNA abundance within tissues, phylogenetic results, presence of Delta-specific monoclonal antibody escape mutants, and high minority SNV richness and diversity indicate potential virus and disease reactivation, leading to the respiratory distress the individual experienced in late November. This aligns with previous observations that higher levels of tissue-specific consensus mutations are found in autopsy cases with longer time intervals between symptom onset and death (3). Although we cannot rule out that a large transmission bottleneck event led to the high variant richness without sampling from the earlier positive tests, estimates of bottleneck sizes for SARS-CoV-2 transmission remain small (14, 21–23). Fluctuations in virus replication of SARS-CoV-2 have been observed in other longitudinal studies of SARS-CoV-2 infections in immunocompromised patients (24), with low levels of virus shedding happening for months before increasing to detectable levels again. Asymptomatic shedding of SARS-CoV-2 RNA may be significantly underestimated, and tissue reservoirs may play an essential role, as individuals with persistent infections are more likely to experience long-COVID systemic symptoms associated with SARS-CoV-2 infections (4, 14, 25). Tissue reservoirs, or compartments that allow continued virus replication, are observed for other RNA viruses, including Ebola virus and human immunodeficiency virus (26, 27). Reactivation of the Ebola virus in reservoirs has also been shown to cause outbreaks within the general population, although the amount of genetic divergence that occurs within reservoirs is limited, likely due to reduced replication of the virus (28, 29). Interestingly, in our data set, dominant sequences with the lowest divergence were found in non-respiratory tissues, including the eyes, appendix, kidney, and tongue.

Most consensus-defining mutations identified were in the spike coding sequence. We detected nine dominant spike haplotypes, four of which were dominant in more than one tissue site. In contrast to previous autopsy studies, which reported limited high-frequency mutations in the spike and RBD (1–3), the 11 spike mutations that define our spike haplotypes are predominately located in the RBD and specifically at amino acid positions where lineage-defining mutations of SARS-CoV-2 variants occur. Among these, two spike substitutions—477N detected at varying frequencies across all tissue sites and 486S, found in ocular tissues, tongue, and the nasal placode—were classified as lineage-defining mutations in later Omicron lineages. Additionally, three RBD substitutions (E406D, G446V, and Y453F) were characterized as emergent mutations in Delta strains following monoclonal antibody treatment and led to weakened neutralization (18). The individual in this study was severely immunocompromised. His underlying CGD, characterized by defects in the NADPH oxidase enzyme complex that impair the phagocytic function of innate immune cells, predisposed him to pulmonary infections that resulted in chronic lung disease with impaired pulmonary structure and function. Daily therapy with sirolimus following HSCT and intermittent oral steroids suppressed his adaptive immune response (30, 31). Although we lack the exact host response data for the individual in this study, altered lung structure and function from CGD may impact SARS-CoV-2 clearance, while the diminished adaptive immune response applies weakened selective pressure on the virus, leading to the high rates of nonsynonymous mutations we observed in our data, especially in the spike region.

We performed protein simulations to understand the structural and functional impacts of the observed combinations of spike substitutions. Most mutant structures increased the predicted structural stability of the spike region and decreased the binding energy required to bind with the ACE2 structure. The Q493K substitution, a change from glutamine, a polar amino acid, to a positively charged lysine residue, was the most influential mutation in terms of binding energy. Q493K is rarely found in circulating strains, but others have also documented reduced IgG antibody recognition of this variant (32). We also performed viral binding and internalization assays on five infectious isolates with distinct spike sequences at two MOIs (0.1 and 1) using TMPRSS2-ACE2 and TMPRSS2 VeroE6 cell lines. Differences between the isolates in binding and internalization efficiencies were mainly observed at an MOI of 0.1 in the ACE2-TMPRSS2 VeroE6 cells.

With the exceptions of S: E191D (stomach), S: P561S (tongue), and N: I157I (right inf. lobe) mutations, consensus mutations observed in one tissue were present as a minor variant in at least one other tissue. This indicates that multiple genotypes likely coexist within the same tissue sites, as further backed by our isolation of multiple infectious viruses from the nasal placode and tongue tissues. Coinfection with different genotypes has important implications in SARS-CoV-2 evolution, as it allows for recombination to occur (33, 34). Interestingly, we rarely observed complete fixation of mutations, which may point to weak bottlenecks or weakened selective pressures within the host that allow for multiple variants to circulate. Except for the tongue, all tissues had three spike substitutions at varying frequencies (V445A, S477N, Q493K). These three substitutions make up the predominant spike haplotype in the sequencing data and were found in four of five infectious isolates. Although we found shared variants across tissue sites, G446V was only in extrapulmonary tissues. Additionally, tracheal, lung, heart, and gastrointestinal tissues all had three nonsynonymous substitutions in the spike (K417R, G476S, K478E) that were not found above our limits of detection in the ocular, nasopharynx, or oropharynx tissue sites, highlighting the tissue-specific differences in the types of mutations maintained. Furthermore, minor variants differed in their genomic location, frequency, and type of mutation depending on the tissue in which they were identified. Additional studies are needed to understand what tissue and cellular factors are shaping these observed differences in viral diversity.

Because we did not observe complete compartmentalization of samples, we calculated pairwise Bray-Curtis dissimilarity indices to quantify the extent of shared low-frequency virus diversity across tissue sites. We found that tissues from the same broad anatomical compartments had lower index scores, suggesting increased mixing. Two distinct clusters formed after hierarchical clustering: one with the ocular, rib, naso-, and oropharynx samples, and another with lower respiratory, gastrointestinal, and heart tissues. Except for the rib sample, the clusters distinguished sites collected from the head versus the chest and abdomen. However, the rib clustered with the one other bone structure, the sinus turbinate. Pulmonary tissues were more likely to share their diversity, which likely reflects how the virus is already well adapted to respiratory epithelium but could also point to extensive tissue damage and a breakdown of compartmentalization within the lungs at the point of collection. The fact that anatomical distance appears to influence the number of shared variants between tissue sites raises interesting questions regarding the routes of SARS-CoV-2 dissemination. One widely accepted theory is that SARS-CoV-2 spreads via the hematogenous route, where the virus may enter the bloodstream via passage through the gastrointestinal tract or respiratory tract and transmit to other organs (1, 8, 9). Thus, hematogenous spread could allow for extrapulmonary-specific viruses to transit back to the respiratory tract and be transmitted to new hosts. In addition, animal studies have shown that intranasal inoculation can lead to the presence of SARS-CoV-2 in the brain, ocular tissues, and lungs, while intratracheal inoculation did not result in the virus entering the brain or ocular tissues, suggesting that the spread to these areas is neuronal and unidirectional (7, 35). SARS-CoV-2 has been identified in the trigeminal neurons in humans and may point to why we observed shared variant populations between the ocular, oropharynx, and nasopharynx tissue sites that were not shared with other tissues. The results presented here offer an important case study, demonstrating high intrahost spike diversity, the coexistence of multiple genotypes within a single tissue site, and tissue-specific differences in virus diversity.

### Limitations of the study

Our study has several limitations, the most notable being that the data were collected from a single individual. However, the inclusion of minor variant data and the extensive tissue set from our study allow us to uniquely bridge observations made by others, including the longitudinal sampling of multiple genotypes from nasopharyngeal swabs during a chronic SARS-CoV-2 infection (20), high spike diversity in immunocompromised patients (34), and tissue compartmentalization of SARS-CoV-2 (1–3). Additionally, we lack host response data, such as antibody titers or gene expression profiles, which would provide insights into the pressures experienced by SARS-CoV-2. Furthermore, virus isolation, binding, and internalization assays were conducted exclusively in VeroE6 cells, which may bias the types of viruses we could isolate and their replication dynamics, particularly those adapted to specific cell and tissue types. Despite these limitations, our data suggest that patients with immunocompromising diseases that weaken adaptive immune responses may create an environment permissive to SARS-CoV-2 evolution and diversification, emphasizing the importance of improving early care strategies for these patients.

## MATERIALS AND METHODS

### Experimental model and study participant details

The study participant was a 57-year-old male patient who died on 28 November 2021 from respiratory failure. An autopsy was performed on 2 December 2021, and tissues were collected as previously described (36) in the National Cancer Institute’s Laboratory of Pathology at the National Institutes of Health Clinical Center following consent of the legal next of kin.

Primary isolation of virus from each tissue was done on VeroE6-TMPRSS2-T2A-ACE2 cells; plaque purification was done on Vero E6-TMPRSS2 cells. Viral binding and internalization efficiencies were tested using VeroE6 cells constitutively expressing human ACE2 and TMPRSS2 (VeroE6-TMPRSS2-ACE2) or only human TMPRSS2 (VeroE6-TMPRSS2).

### Autopsy, RNA isolation, SARS-CoV-2 RNA, and subgenomic RNA quantification

Tissues were collected from the patient as previously described, with adjacent portions flash-frozen and preserved in RNAlater (Invitrogen) (1). SARS-CoV-2 RNA was isolated using the RNeasy Mini, RNeasy Fibrous Tissue Mini, RNeasy Lipid Tissue Mini, and QIAamp Viral RNA Mini kits (Qiagen) according to the manufacturer’s protocols. SARS-CoV-2 RNA quantification was performed using the QX200 AutoDG ddPCR system (Bio-Rad) as detailed in Stein et al. (1). Averages of the N1 and N2 technical replicates are reported in Table S1. SARS-CoV-2-positive samples are those with an average greater than or equal to the manufacturer’s limit of detection of 0.1 copies per microliter and ≥2 positive droplets per well. Subgenomic RNA analyses were performed on 32 tissues using 5 µL of sample RNA input into a one-step real-time RT-qPCR assay targeting the envelope (E) gene.

### Amplification, library preparation, and sequencing

SARS-CoV-2 RNA was amplified using the ARTIC V4 primer set and protocol. The ~400 bp amplicons were cleaned using AMPure beads and used as input into the Illumina DNA Prep kit (Illumina, San Diego, CA) according to the manufacturer’s protocol. The final concentration and average fragment size of each library were quantified using the Qubit dsDNA HS Assay (Thermo Fisher Scientific, Waltham, MA) and a high-sensitivity D1000 ScreenTape (Agilent, Santa Clara, CA), respectively. Libraries were diluted to be equimolar and pooled at a final concentration of 4 nM before sequencing on the MiSeq (Reagent Kit v.3, 600 cycles, Illumina, San Diego, CA). Amplification, library preparation, and sequencing for all extractions, including vRNA from isolates, were performed twice using vRNA from the same extraction.

Where available, vRNA from the tissue extractions was also sequenced using PacBio HiFi Viral protocol as recommended by the manufacturer (Pacific Biosciences) and outlined previously in Nicot et al. (16). In short, SARS-CoV-2-specific RNA was enriched with a panel of highly specific probes. Following capture, these probes were used to prime molecular inversion PCR to generate tiled amplicons of ~800 bp (~650 bp target, combined ~150 bp for flanking tags) across the SARS-CoV-2 genome. A second round of amplification was performed to introduce sequencing barcodes to each sample to allow for sample multiplexing. Amplified viral genome material from all samples was equimolar pooled and used as input into SMRTbell template preparation, performed as recommended by the manufacturer. Briefly, the amplicon pool underwent DNA damage repair, end repair, and ligation to hairpin sequencing adapters. Endonuclease treatment following adapter ligation was used to remove any unligated amplicon material that would interfere with successful library loading. Libraries were then purified with AMPure PB beads (Pacific Biosciences, Menlo Park, CA), and quality and quantity were evaluated using the Fragment Analyzer (Agilent Biosciences, Santa Clara, CA) and Qubit Fluorometer 4 (Thermo Fisher Scientific, Waltham, MA), respectively. Sequencing was performed using a single pool for all autopsy samples on the Sequel IIe system using polymerase v.2.1 and 8 h movies. Circular consensus reads with accuracy greater than 99% (high fidelity, “HiFi” reads) were generated on instrument and used for all downstream analyzes.

### Alignments, filtering, consensus sequences, and variant calling

Primers and low-quality nucleotides were trimmed from the raw sequencing data using Trimmomatic (v.0.39) before aligning the reads to the SARS-CoV-2 reference genome (NC_045512.2, Wuhan-1) using BWA-MEM (v.0.7.17) (37–39). Duplicate reads were removed from the alignments using PicardTools (v.2.22.2) RemoveDuplicates function (40). Given the sparse read coverage observed in the ARTIC V4 Illumina data, the replicate short-read (average read length: 150 bp, Illumina) data for each sample were combined into one file using Samtools (v.1.14) before calling SNVs using timo (41, 42). Sample consensus sequences were generated from the timo outputs for all the long-read (average read length: 650 bp, PacBio) alignments and the short-read (Illumina) alignments for the tongue and pericardium samples. Samples were required to have at least 80% of the genome covered at 10× read depths to be used for consensus analyses (Table S1).

The SARS-CoV-2 consensus sequences were defined as an AY.119 Delta lineage using NextClade (v.3.8.2) (43). Therefore, all single-nucleotide variants identified were compared to an AY.119 reference sequence while maintaining the NC_045512.2 reference genome numbering (i.e., ignoring deletions and insertions). Mutations in the 5´ and 3´ untranslated regions of the viral genome were ignored due to poor sequencing coverage in both the short-read and long-read data. Due to sparse and uneven short-read sequencing, SNV analyses were performed using long-read data and the short-read data for the tongue and pericardium data (Table S1). Consensus, or major, variants were considered those with the highest relative frequency at positions with ≥10× read depths. Minor variants were considered nucleotide variants with the lowest relative frequency at a given position. All variants found in the long-read data at frequencies of 5%–100% and a read depth of ≥100× were kept for analyses and confirmed to be present in the short-read data (≥2%, 200×) when read depths in the short-read libraries were ≥10×. Variants found in both the long-read (2%–5%, 100×) and short-read data (≥2%, 200×) were also kept for analyses.

### Circulating variants and lineage-defining mutations

Proportional data for SARS-CoV-2 lineages and variant positions circulating in the USA from 1 January 2021 to 1 January 2024 were downloaded from CoV-Spectrum (44) (cov-spectrum.org) and visualized in R (45) (v.4.2.3) using ggplot2 (3.4.3) (46).

### Phylogenetic analyses

The 27 SARS-CoV-2 consensus sequences from samples with at least 10× read depths across 80% of the genome were used for phylogenetic analyses. Complete consensus sequences of 6,000 AY.119 strains circulating in humans in the USA from 1 August 2021 to 31 January 2022 were randomly selected and downloaded from GISAID (https://doi.org/10.55876/gis8.240924yd) (47). All sequences were aligned to the NC_045512.2 (Wuhan-1) reference sequence with the 5´ (1–265) and 3´ (29,675–29,903) removed using MAFFT (v.7.475) and input parameters: --6merpair --keeplength and --addfragments (48). Maximum-likelihood trees were constructed with IQ-TREE (v.2.2.0.5) using the generalized time-reversible (GTR+G) model and 1,000 bootstrap replicates (49). Substitution rates were estimated using the time-resolved data from TreeTime (v.0.11.1) with collection dates for the 6,000 GISAID provided as input with the maximum-likelihood tree generated using IQ-TREE (50). Outliers were removed from the tree and root-to-tip distance calculations. Root-to-tip calculations were performed using ape (v.5.7-1), and tree visualization was done in R (4.2.3) using ggtree (3.6.0) and treeio (v.1.22.0) (51–53).

### Haplotype construction

SARS-CoV-2 spike haplotypes were assembled for each tissue site using CliqueSNV (v.2.0.3). CliqueSNV assembles minority SNVs into predicted haplotypes by calculating the probability that SNV pairs are linked using read counts, relative frequencies, and haplotype length. We limited the haplotype region to the spike coding region (21,563–25,384) and set the haplotype frequency and read depth parameters to 10% and 5× to increase confidence (17). All positions were compared to the AY.119 reference with NC_045512.2 (Wuhan-1) numbering.

### Bray-Curtis dissimilarity index

To compare the minor SNV populations across samples, the pairwise BCI were calculated by pulling each sample’s relative frequency of SNVs (0%–100%) that were found as a minor variant in at least one sample. Samples that did not have the SNV above our thresholds were marked as having the SNV at 0%. Those found only as a consensus variant (>50%) in tissue sites were not used, as we wanted to see the flow of variants and diversity found at low relative frequencies in tissues. Nucleotide variants were considered independent.


$${BC}_{jk}=1-\frac{2C_{jk}}{S_{j}+S_{k}}$$

The BCI was calculated between two tissue sites (*j* and *k*), where *C_jk_* is the sum of the lesser frequencies of each minor variant between tissue sites *j* and *k,* and *S_j_* and *S_k_* are the total sums of minor variant frequencies at each respective tissue site.

### Minor variant richness, divergence, and dN/dS

Richness of minor variants was calculated by counting the total number of single-nucleotide variants for each sample that passed our required confidence thresholds (outlined above). Divergence per site was calculated by summing the relative frequency of either nonsynonymous or synonymous substitutions and normalizing by the total number of nonsynonymous or synonymous sites in the coding regions of the genome (54). Similarly, dN/dS ratios were calculated by taking the ratio of the number of nonsynonymous mutations normalized by the total number of nonsynonymous sites in the coding regions of the genome to the number of synonymous mutations normalized by the total number of synonymous sites in the coding regions of the genome. For both calculations, deletions, insertions, and non-coding or untranslated sites were not included, and all variant positions were considered independent and unlinked, as confidence in linked sites decreases for low-frequency SNVs.

### Spike structure remodeling and validation

The open spike crystallized structures and protein sequences of the SARS-CoV-2 spike were downloaded from the PDB for Wuhan-01 (PDB ID: 7CAK) and Delta AY.119 (PDB ID: 7W98) (55). Due to missing residues in the three-dimensional (3D) structure, remodeling was done using the SWISS-MODEL server (56). Initially, the template structure was identified, followed by template-target sequences alignment using MAFFT (48) alignment tool. The reliability of the best three resulting models was assessed using PROCHECK (57), VERIFY3D (58), and ProSA-web (59) tools. The best structure was used for subsequent analyses. The consensus evaluation of the remodeled reference structure (PDB ID: 7W98) indicated a reliable and accurate starting structure, with 81.86% of residues scoring ≥0.1 in the 3D/1D profile, an overall quality factor >87.3%, and stereochemistry parameters showing 88.8% of residues in core regions. Mutant residues for each spike haplotype were manually inserted at their specified positions using Discovery Studio Visualizer (60). The protonation state of pH 7.0 was applied to all systems using the H^++^ Server (http://biophysics.cs.vt.edu/H++) (61).

### Atom-wise molecular dynamics (MD) simulations

A total of 80 ns all-atom MD simulations were performed using GROningen Machine for Chemical Simulations (GROMACS v.2023) (62) on the NIH Biowulf HPC for Wuhan-01, Delta-AY.119, and 13 mutant spike sequences (S: G1267R could not be simulated). Each structure topology was generated, submerged in a dodecahedron box (2.0 Å spaced between each edge of the box and protein), and solvated in TIP3P solvent model (63). Following solvation, counterions (sodium and chloride at 0.15 M concentration) were added to neutralize the system’s charge. Energy minimization was then performed to relax steric clashes and relieve unfavorable contacts. Subsequently, equilibration was achieved in two phases—NVT (constant number of particles, volume, and temperature) and NPT (constant number of particles, pressure, and temperature) at reference temperature of 300 K—allowing the system to reach a stable state. The 80 ns MD run utilized the leap-frog integration algorithm and periodic boundary conditions to model a representative section of the system. Long-range electrostatic interactions were treated with the Particle Mesh Ewald method (64). Hydrogen bonds were constrained across all systems. Trajectory data were saved for subsequent analysis, including overall RMSD, RMSD of subunits and domains of the spike protein (S1, S2, RBD, NTD), and the ACE2 receptor, and RMSF. The MD trajectories were visualized using Visual Molecular Dynamics (65). Binding energy contributions between spike (atom 1–17,457) and the human receptor ACE2 (atom 17,458–26,974) were computed using gmx_mmpbsa over the last 10 ns of simulation across all systems (66). gmx_mmpbsa uses molecular mechanics Poisson-Boltzmann surface area to calculate free energies by incorporating the GROMACS files into the following equation:


$$\Delta G_{binding}=\Delta E_{MM}+\Delta G_{solvation}-T\Delta S$$

where Δ*G*_binding_ is the total binding free energy, Δ*E*_MM_ represents the molecular mechanics (van der Waals and electrostatic interactions), Δ*G*_solvation_ indicates polar and non-polar contributions, and *T*Δ*S* shows the entropic contribution where *T* stands for temperature, and Δ*S* is the change in entropy upon binding. For the spike-ACE2 complex, each component was calculated individually. The total delta energy terms were further decomposed to highlight the contribution of each mutant residue.

### Virus isolation

Virus isolation was performed on VeroE6-TMPRSS2-T2A-ACE2 cells grown in DMEM containing 10% FBS, 50 U/mL penicillin, 50 µg/mL streptomycin, and 10 µg/mL puromycin as described previously (1). Briefly, tissues were homogenized in 1 mL DMEM and used to inoculate cells in a 24-well plate with 250 µL of cleared homogenate and a 1:10 dilution thereof. Plates were centrifuged for 30 min at 1,000 rpm followed by 30 min incubation at 37°C and 5% CO_2_. The inoculum was then removed and replaced with 500 µL DMEM containing 2% FBS, 50 U/mL penicillin, and 50 µg/mL streptomycin. Six days after inoculation, the cytopathic effect (CPE) was scored. A blind passage of samples where no CPE was observed was performed according to the same method. Supernatant from samples where CPE was observed was harvested and stored at −70°C. Presence of SARS-CoV-2 was confirmed by qRT-PCR to detect E sgRNA. Plaque purification was done on VeroE6-TMPRSS2 cells seeded at a density of 1.25 × 10^6^ cells per well in six-well tissue culture plates. The following day, the medium was removed and replaced with 200 µL of serially diluted tissue samples in DMEM supplemented with 1% FBS. After incubation for 1 h at 37°C, 2 mL of agarose overlay supplemented with DMEM with 5% FBS was added. Plates were incubated for 48 h, and 12 plaques were picked from each sample and inoculated in 12-well plates. Three to five plaque-purified viruses from each sample were sent for sequencing as previously described above.

### Viral binding and internalization assays

Five plaque-purified isolates (Table S2) were selected to phenotypically test their binding and internalization efficiencies using VeroE6 cells constitutively expressing human ACE2 and TMPRSS2 or only human TMPRSS2 at MOIs of 1 and 0.1. To assess virus binding to the cell surface, cells were incubated with either 0.1 or 1 MOI of relevant viral isolate on ice for 1 h at 37°C before washing three times with cold PBS. After washing, 600 µL of RNA lysis buffer was added directly to the cells. To determine viral internalization into the cells, cells were incubated with the desired MOI (0.1 or 1) of viral isolates on ice for 1 h at 37°C. The cells were washed three times with cold PBS to remove any virus that did not bind to the cells. Pre-warmed 2 mL of serum-free DMEM medium was added to the cells, followed by a 3 h incubation at 37°C. The cells were washed three times with PBS, followed by addition of 600 µL RNA lysis buffer to the cells. The RNA extractions were performed on all harvested samples using Direct-Zol RNA Miniprep Kit (Zymo), and cDNA was prepared using High-Capacity cDNA Reverse Transcription Kit (Thermo Fisher Scientific) as per the manufacturer’s protocol. Viral RNA levels and replication were measured as previously described in Kar et al. (67). Briefly, qRT-PCR was performed using IDT Prime Time gene expression master mix on a QuantStudio5 qPCR system using the cycling conditions recommended by the manufacturer. To measure viral RNA levels, SARS-CoV-2 RdRp-specific forward primer: GTGARATGGTCATGTGTGGCGG; reverse primer: CARATGTTAAASACACTATTAGCATA, and probe 56-6-carboxyfluorescein [FAM]/CAGGTGGAA/ZEN/CCTCATCAGGAGATGC/3IABkFQ were used. Ct values were normalized to the reference gene GAPDH and represented as a fold change over values from time-matched mock samples.

### Quantification and statistical analysis

All statistical analyses and data visualizations were performed using R and ggplot2. Welch’s *t*-test was used to assess the significance of minor variant comparisons (richness, divergence, and dN/dS) and isolate comparisons. Pearson’s correlation coefficient was used to evaluate the strength and significance of correlations between variables. Differences in RMSD values between AY.119 and all mutant spike haplotypes were analyzed using the Kruskal-Wallis test. Changes in Gibbs free energy were compared using one-way ANOVA. A *P*-value of less than 0.05 was considered significant for all tests, while ns indicates comparisons that were not significant. The specific statistical tests and *P*-values are detailed in the results text and figure legends.

## Data Availability

Viral isolates from binding and internalization cell culture experiments are available upon request. Raw sequencing data and consensus sequences are available under the Bioproject PRJNA1208042. Code, variant data files, and binding and internalization data files used for analyses are available on GitHub at https://github.com/GhedinSGS/SARS-CoV-2_TissueDiversity and Zenodo (https://doi.org/10.5281/zenodo.14790788). Additional data reported in this paper that are needed for reanalysis can be requested from the lead contact.

## SUPPLEMENTAL MATERIAL

Fig. S1 jvi.00857-25-s0001.pdf Sequencing quality control.

Fig. S2 jvi.00857-25-s0002.pdf Frequency and read depth of variants.

Fig. S3 jvi.00857-25-s0003.pdf Dynamics of SARS-CoV-2 VOC spike mutations.

Fig. S4 jvi.00857-25-s0004.pdf Measurements of stability of the spike subunits and ACE2.

Fig. S5 jvi.00857-25-s0005.pdf Relative frequencies of new consensus variants from infectious isolates.

Fig. S6 jvi.00857-25-s0006.pdf Dynamics of tissue specific minor variants.

Supplemental tables jvi.00857-25-s0007.pdf Tables S1 to S3.

ASM does not own the copyrights to Supplemental Material that may be linked to, or accessed through, an article. The authors have granted ASM a non-exclusive, world-wide license to publish the Supplemental Material files. Please contact the corresponding author directly for reuse.
