# Supplementary material for: Tissue tropism and functional adaptation of the SARS-CoV-2 spike protein in a fatal case of COVID-19
Source: J Virol. 2025 Oct 31;99(11):e00857-25. doi: 10.1128/jvi.00857-25 (PMC12645954; doi:10.1128/jvi.00857-25)
Supplement: Fig. S1 — Sequencing quality control. [file jvi.00857-25-s0001.pdf]

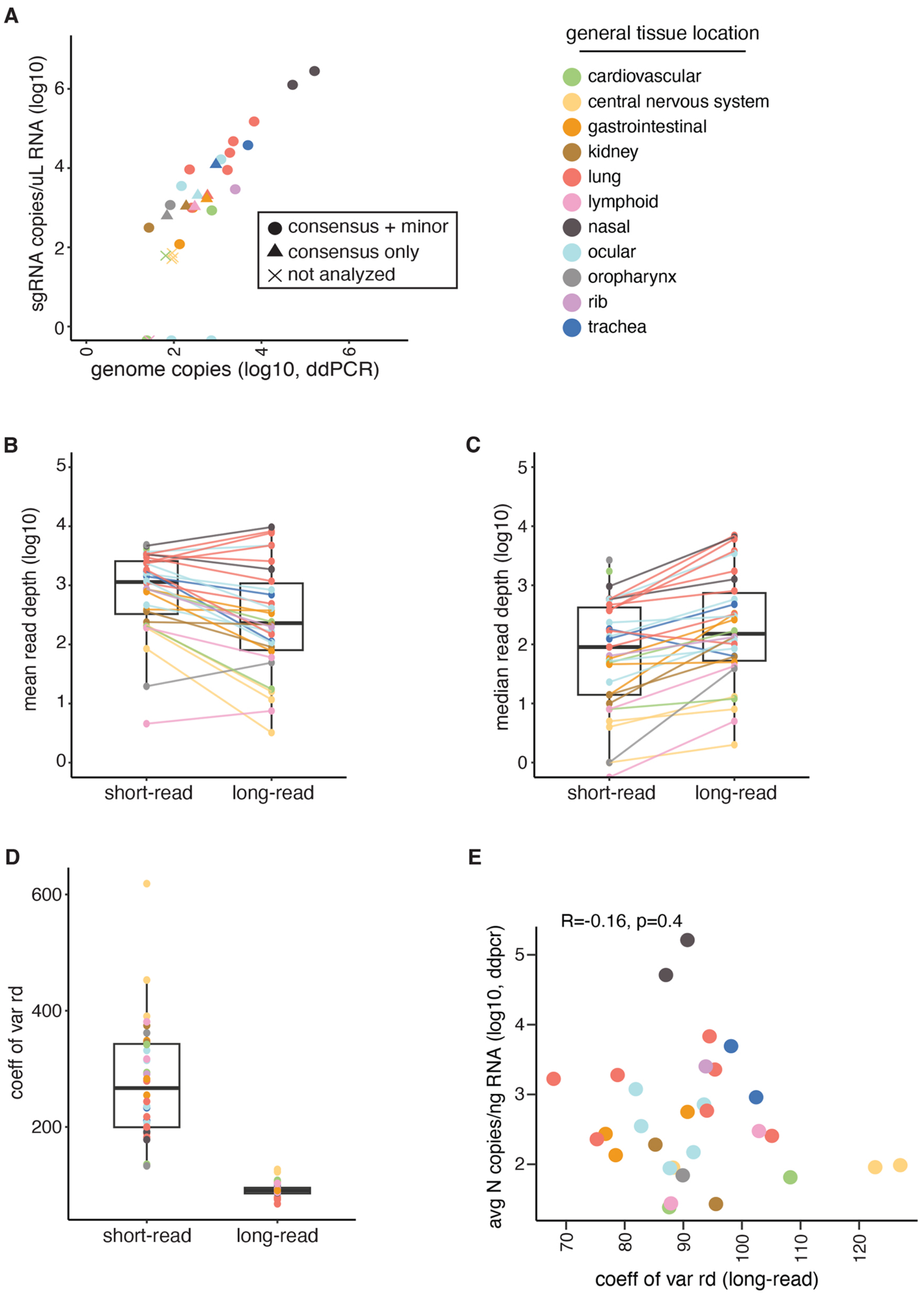

Figure S1. Sequencing quality control, related and Table S1. (**A**) Log_{10} N gene copies/ng RNA (x-axis) versus the log_{10} subgenomic RNA (sgRNA) copies/microliter (uL) of RNA (y-axis). Point shape represents whether the sample was used for all (consensus and minor) variant analyses (circle), consensus analyses only (triangle) or not used for variant analyses (cross). (**B-C**) Boxplots showing the distribution of (**B**) log_{10} mean and (**C**) log_{10} median read depth for each sample (points) sequenced using either short-read (Illumina) (n=32) or long-read (PacBio) (n=30) (x-axis) sequencing platforms. The color of each point indicates the general tissue location and is maintained throughout the figure. (**D**) The distribution of the coefficient of variation (CV) of read depth (rd) [CV = (rd sd/rd mean) *100] for each sample sequenced using either short-read or long-read data (x-axis). Al boxplots represent the median (middle), first and third quartiles (box), and 1.5*interquartile range (whiskers). (**E**) Log_{10} mean number of nucleocapsid (N) gene copies/nanogram (ng) of RNA (y-axis) versus the long-read CV of rd (x-axis). The Pearsons correlation and p-value are provided in the top left corner.
