# Supplementary material for: Tissue tropism and functional adaptation of the SARS-CoV-2 spike protein in a fatal case of COVID-19
Source: J Virol. 2025 Oct 31;99(11):e00857-25. doi: 10.1128/jvi.00857-25 (PMC12645954; doi:10.1128/jvi.00857-25)
Supplement: Fig. S2 — Frequency and read depth of variants. [file jvi.00857-25-s0002.pdf]

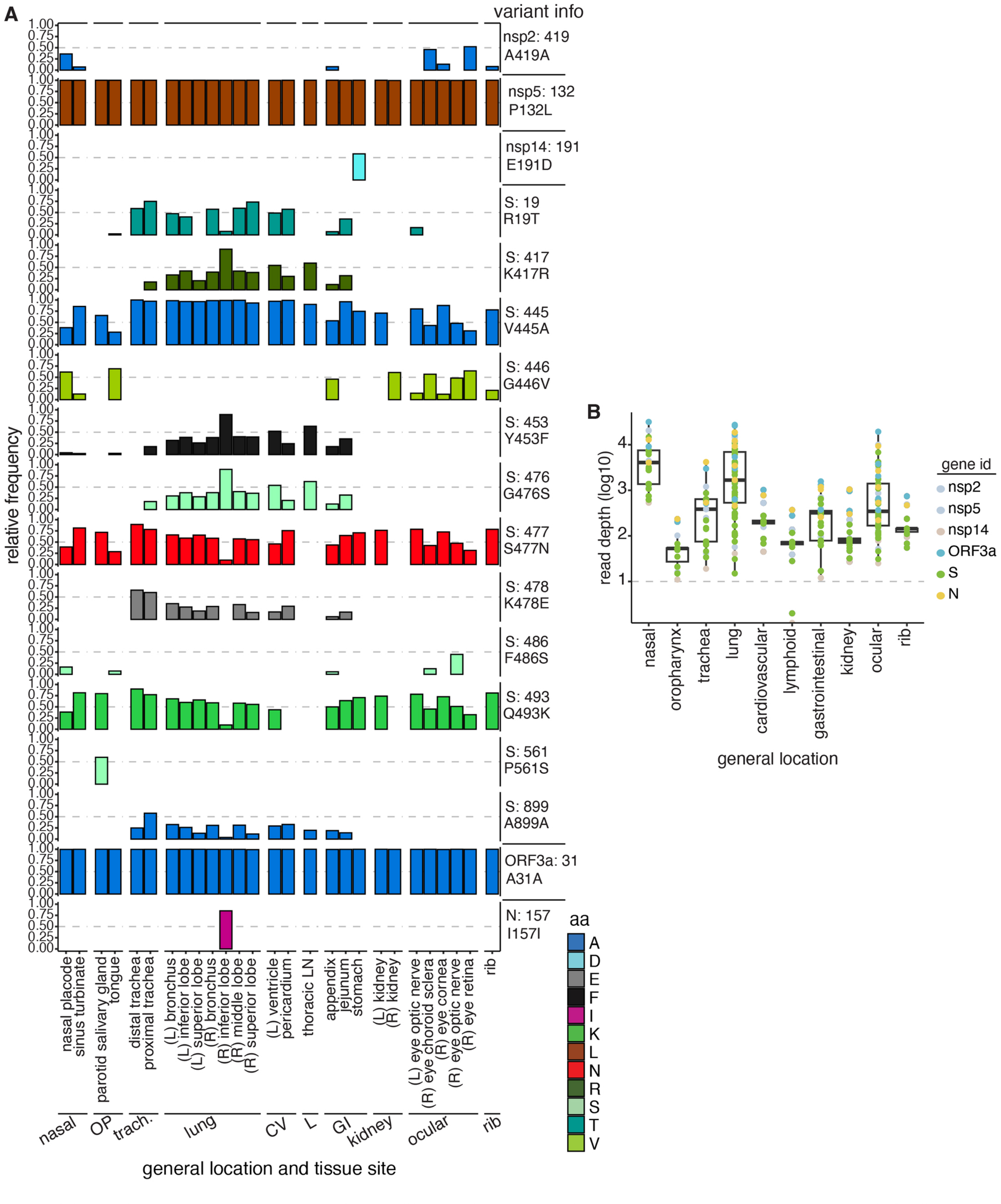

Figure S2. Frequency and read depth of variants at sites with consensus mutations, related to Table 1 and Figure 2. (**A**) Relative frequency (y-axis) of variants across the different tissue sites (x-axis). Data are grouped by general tissue location across and coding region, amino acid position, and variant amino acid going down. Colors indicate the amino acid of the variant. Only variants found at ≥5% at genomic sites with ≥100X read depth and at position where a consensus mutation was identified in the dataset are shown. Horizontal gray dashed lines represent the 50% frequency line. (**B**) Log_{10} read depth of variant positions across the general tissue locations. Each point represents the read depth at a variant position outlined in (A) for each tissue site. Color represents the coding region the variant is located in. The dashed horizontal gray line indicates the 10X read depth requirement for confidently calling the major or consensus nucleotide for a sample. NSP: non-structural protein, ORF: open reading frame. S: spike, N: nucleocapsid.
