# Supplementary material for: Tissue tropism and functional adaptation of the SARS-CoV-2 spike protein in a fatal case of COVID-19
Source: J Virol. 2025 Oct 31;99(11):e00857-25. doi: 10.1128/jvi.00857-25 (PMC12645954; doi:10.1128/jvi.00857-25)
Supplement: Fig. S3 — Dynamics of SARS-CoV-2 VOC spike mutations. [file jvi.00857-25-s0003.pdf]

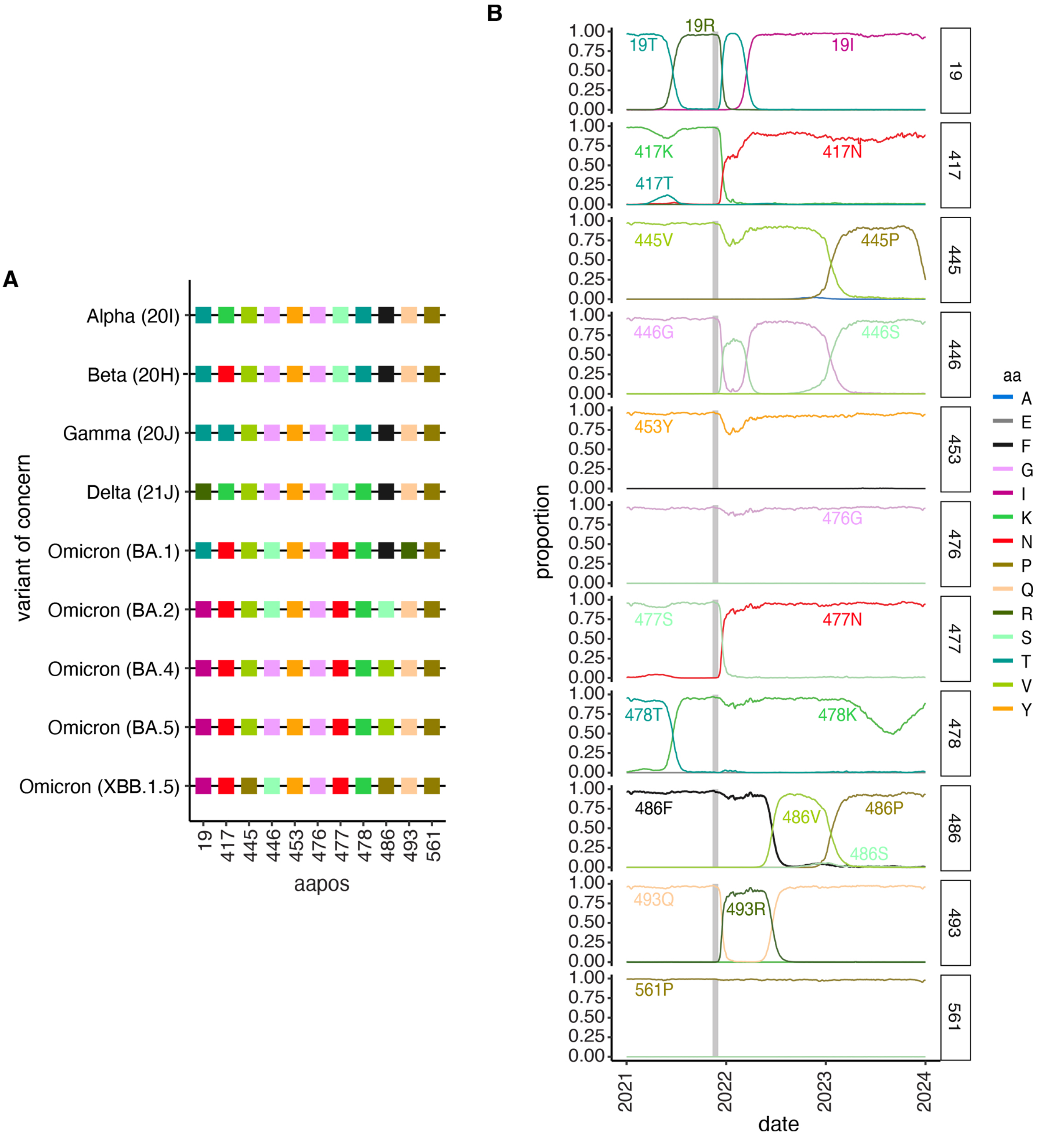

Figure S3. Dynamics of SARS-CoV-2 variant of concern spike mutations, related to Table 1 and Figure 2. (**A**) The consensus amino acid residues at each spike residue position (x-axis) for variants of concern circulating in the United States from January 1st, 2021 – January 1st, 2024. The clade (NextClade) or PANGO lineage (Omicron only) is also provide for each variant of concern. (**B**) The relative proportion of different amino acid mutations within the spike region of strains circulating in the United States from Jan. 1st, 2021 – Jan. 1st, 2024. Only spike positions found to be different between the autopsy tissue sites were pulled. The gray box highlights the 3-week period surrounding the patient's second positive COVID-19 test (November 12, 2021 – December 03, 2021). Color indicates the amino acid residue and is consistent throughout the figure.
