# Supplementary material for: Tissue tropism and functional adaptation of the SARS-CoV-2 spike protein in a fatal case of COVID-19
Source: J Virol. 2025 Oct 31;99(11):e00857-25. doi: 10.1128/jvi.00857-25 (PMC12645954; doi:10.1128/jvi.00857-25)
Supplement: Fig. S4 — Measurements of stability of the spike subunits and ACE2. [file jvi.00857-25-s0004.pdf]

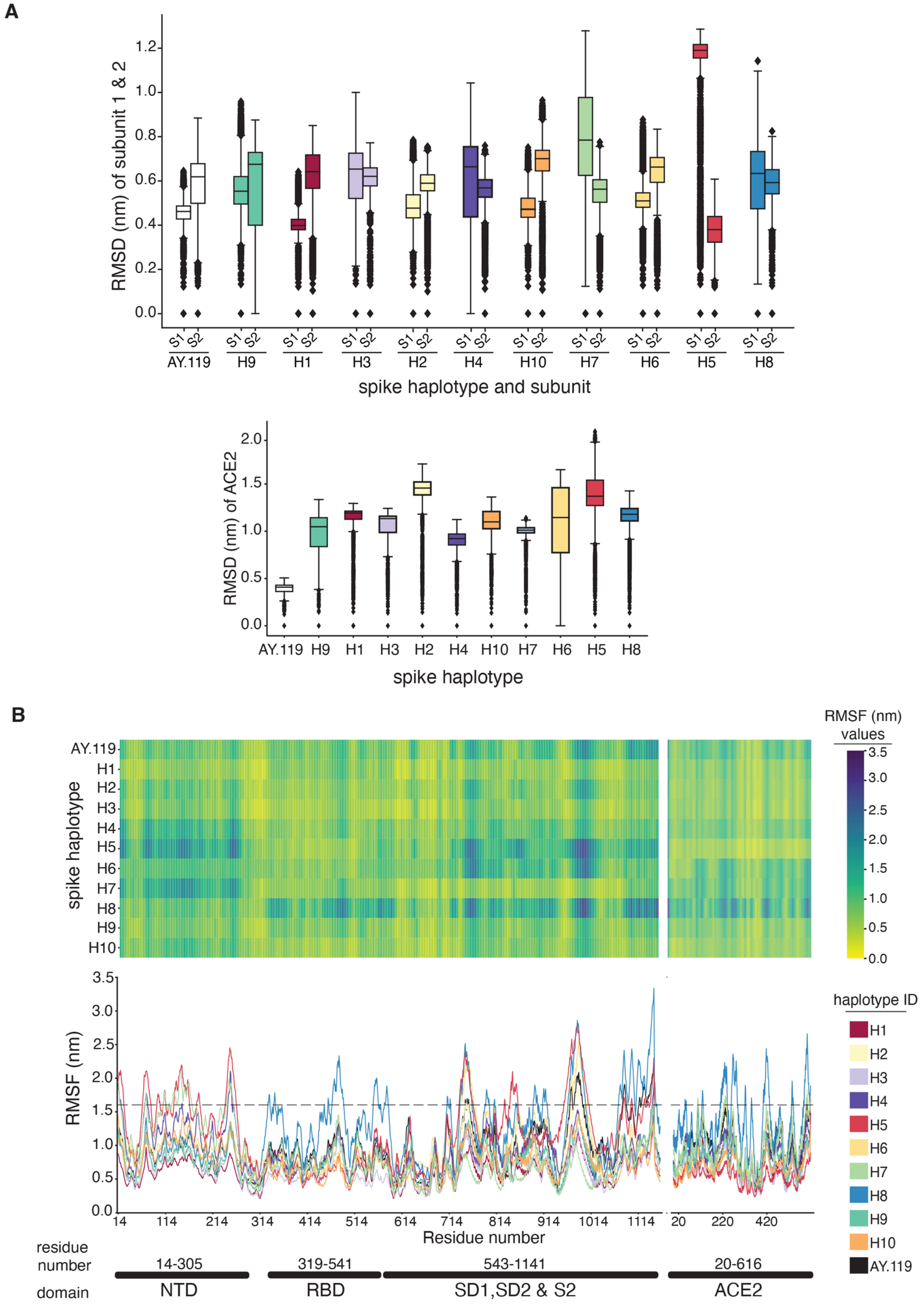

Figure S4. Measurements of stability of the spike subunits and ACE2, related to Figure 3. (**A**) The distribution of the mean RMSD values (y-axis) for the spike subunit 1 (S1) and 2 (S2) (**top**) and the ACE2 binding region (**bottom**) for the spike-ACE2 complex across spike sequences (x-axis). Average RMSD values were calculated for 100 individual frames at every nanosecond (ns) of an 80ns simulation (n=8000). Color indicates the haplotype sequence and is consistent across all figures. Boxplots represent the median (middle line), first and third quartiles (box), 15 * interquartile range (whiskers), and outliers (diamond points). (**B**) Residue fluctuations within the spike-ACE2 complex. A heatmap (**top**) and line plot (**bottom**) of root mean square fluctuations (RMS) in nanometers (nm) for each residue (x-axis) in the spike-ACE2 complex. Complex domains and their residue positions are outlined at the bottom. Line colors represent the mutant spike haplotype sequence (H1-H10) or the AY. 119 reference sequence (black). The horizontal dashed line (bottom) indicates the mean RMS across all sequences and residues.
