# Supplementary material for: Tissue tropism and functional adaptation of the SARS-CoV-2 spike protein in a fatal case of COVID-19
Source: J Virol. 2025 Oct 31;99(11):e00857-25. doi: 10.1128/jvi.00857-25 (PMC12645954; doi:10.1128/jvi.00857-25)
Supplement: Fig. S5 — Relative frequencies of new consensus variants from infectious isolates. [file jvi.00857-25-s0005.pdf]

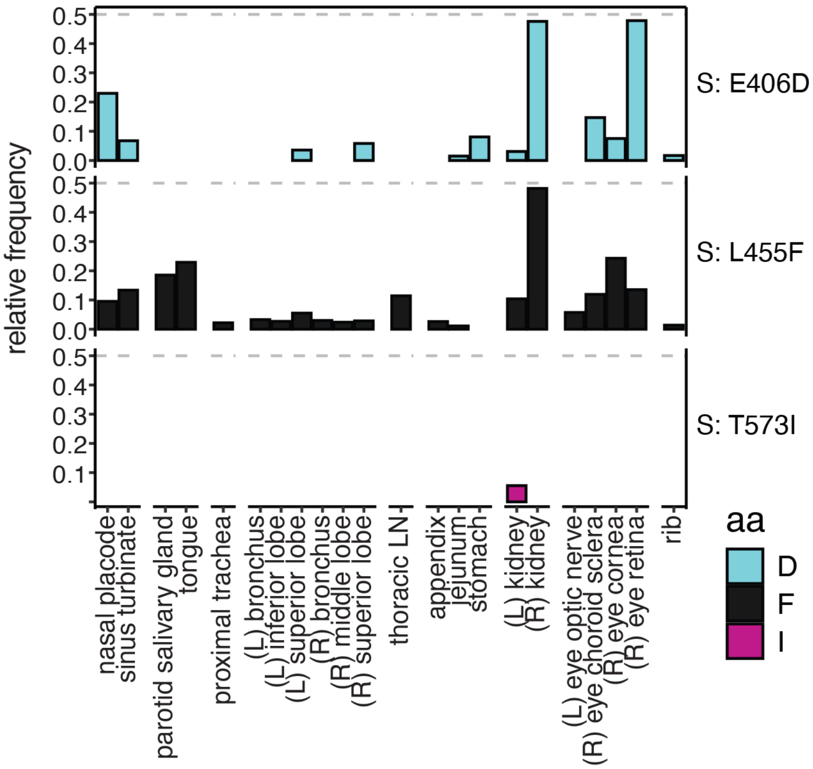

Figure S5. Relative frequencies of new consensus variants identified in infectious isolates, related to Figure 4 and Tables S2 and S3. Relative frequency (y-axis) of mutations across the different tissue sites (x-axis) of variants found as a consensus mutation in the infectious isolates but not the original sequencing data using low-stringency cutoffs (≥1%, 50X). Data are grouped by general tissue location across and coding region, amino acid position, and variant amino acid going down. Colors indicate the amino acid of the variant and the horizontal gray dashed line highlights 50% (0.50) frequency.
