# Supplementary material for: Tissue tropism and functional adaptation of the SARS-CoV-2 spike protein in a fatal case of COVID-19
Source: J Virol. 2025 Oct 31;99(11):e00857-25. doi: 10.1128/jvi.00857-25 (PMC12645954; doi:10.1128/jvi.00857-25)
Supplement: Fig. S6 — Dynamics of tissue specific minor variants. [file jvi.00857-25-s0006.pdf]

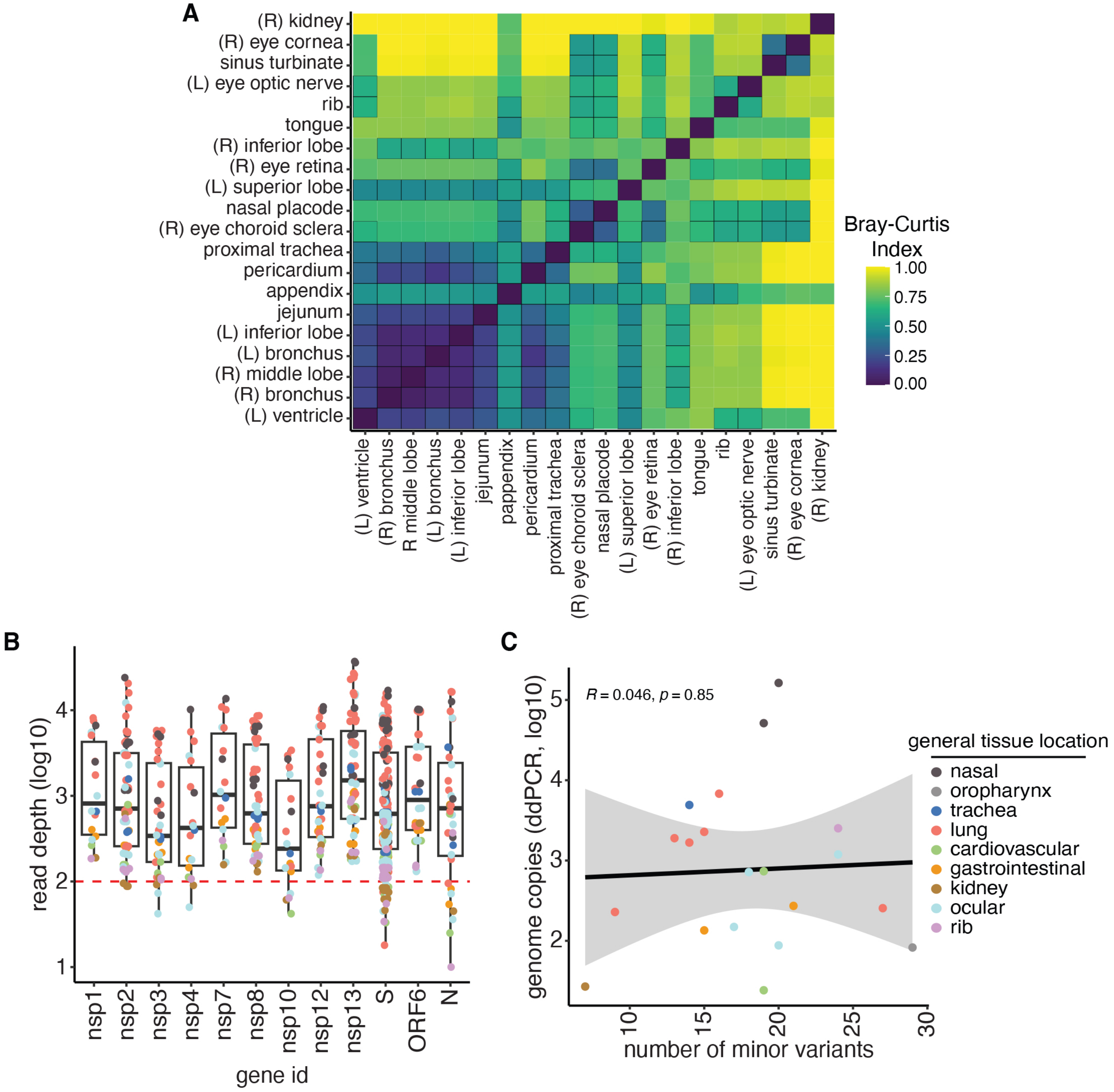

Figure S6. Dynamics of tissue specific minor variants, related to Figure 5. (**A**) A heatmap of the pairwise Bray-Curtis index calculations for each tissue site. Color indicates the Bray-Curtis index score. BCI values assume independence between variants and were calculated using positions where a variant was present as a minor variant in at least one sample. (**B**) The log_{10} read depth for each tissue site at defining variant positions outlined in **Figure 5D**. Each point represents an individual tissue site, and color indicates the general location of the tissue. The red dashed horizontal line indicates the necessary read depth for calling a minor variant. NSP: non-structural protein, S: spike, ORF: open reading frame, N: nucleocapsid. (**C**) The number of minor single nucleotide variants across the genome (x-axis) versus the log_{10} mean number of nucleocapsid (N) gene copies/nanogram (ng) of RNA (y-axis). Each point represents an individual tissue site, and color indicates the general anatomical location where the tissue is located.
