# Supplementary material for: Tissue tropism and functional adaptation of the SARS-CoV-2 spike protein in a fatal case of COVID-19
Source: J Virol. 2025 Oct 31;99(11):e00857-25. doi: 10.1128/jvi.00857-25 (PMC12645954; doi:10.1128/jvi.00857-25)
Supplement: Supplemental tables — Tables S1 to S3. [file jvi.00857-25-s0007.pdf]

Table S1: Tissue sites collected from autopsy

tissue site	ddPCR		subgenomic RNA		variant analyses	
	Avg. N copies/ng RNA	log10 avg. N copies/ng RNA	Cq	copies/µL RNA	consensus	minor
**nasal placode	162918.48	5.21	21.43	2,815,778.34	X	X
**sinus turbinate	51393.34	4.71	22.69	1,264,954.15	X	X
**L inferior lobe	6799.58	3.83	26.06	150,147.41	X	X
**proximal trachea	4918.07	3.69	28.24	37,860.93	X	X
**rib	2520.86	3.40	32.28	2,918.54	X	X
**L bronchus	2274.81	3.36	27.88	47,537.85	X	X
**R middle lobe	1897.79	3.28	28.93	24,385.00	X	X
**R bronchus	1673.74	3.22	30.51	8,965.51	X	X
**L eye - Optic Nerve	1191.00	3.08	29.54	16,590.76	X	X
**distal trachea	911.69	2.96	30.01	12,293.56	X	--
*pericardium	734.75	2.87	34.23	852.6	X	X
**R eye - choroid/sclera	718.08	2.86	--	--	X	X
**R superior lobe	585.81	2.77	32.85	2,031.09	X	X
**stomach	561.87	2.75	33.15	1,684.51	X	--
**R eye - optic nerve	351.66	2.55	32.84	2,053.41	X	--
**thoracic lymph node	299.02	2.48	33.88	1,064.09	X	--
**appendix	272.30	2.44	33.98	998.25	X	X
**L superior lobe	255.06	2.41	33.98	997.12	X	--
**R inferior lobe	228.46	2.36	30.46	9,226.86	X	X
**L kidney	191.02	2.28	33.83	1,092.32	X	--
**R eye - cornea	148.98	2.17	31.98	3,528.98	X	X
**jejunum	134.98	2.13	37.32	119.81	X	X
**dura matter	97.06	1.99	38.55	55.3	--	--
**basilar artery	90.76	1.96	38.13	71.77	--	--
**cervical spinal cord	89.44	1.95	38.69	50.44	--	--
**R eye - retina	87.79	1.94	--	--	X	X
*tongue	82.53	1.92	33.73	1,168.11	X	X
**parotid salivary gland	69.45	1.84	34.73	620.68	X	--
**thoracic aorta	64.99	1.81	38.38	61.4		
L eye - choroid/sclera	50.22	1.70				
abdominal aorta	36.11	1.56				
**spleen	27.50	1.44	--	--		
**R kidney	26.94	1.43	35.81	312.97	X	X
**L ventricle	24.22	1.38	--	--	X	X
L eye - cornea	22.54	1.35				

L eye - lens	**21.46**	**1.33**		
ileum	**18.79**	**1.27**		
pancreas	**15.05**	**1.18**		
IV septum	**14.08**	**1.15**		
R ventricle	**13.22**	**1.12**		
L adrenal gland	**10.84**	**1.03**		
colon	**9.99**	**0.999**		
skeletal muscle	**8.46**	**0.928**		
sciatic nerve	**8.15**	**0.911**		
duodenum	**7.72**	**0.887**		
carotid body	**7.49**	**0.875**		
testis	**4.71**	**0.673**		
carotid artery	**2.70**	**0.432**		
esophagus	**2.19**	**0.340**		
blood clot - aorta	**2.18**	**0.338**		
thyroid	**1.92**	**0.284**		
L eye - retina	**1.81**	**0.258**		
R eye - lens	**1.62**	**0.210**		
vena cava	**1.48**	**0.171**		
tracheal lymph node	**1.07**	**0.028**		
liver	**1.03**	**0.014**		
femoral nerve	**0.7016**	**-0.154**		
R adrenal gland	**0.6370**	**-0.196**		
Mesenteric lymph node	**0.4631**	**-0.334**		
bronchial lymph node	**0.3178**	**-0.498**		
skull	**--**	**--**		
skin - healthy	**--**	**--**		
occipital lobe	**--**	**--**		
skin - lesion R forearm	--	--		
thymus	--	--		
frontal lobe	--	--		
parietal lobe	--	--		
temporal lobe	--	--		
midbrain	--	--		
cerebellum	--	--		
thalamus	--	--		
hypothalamus	--	--		
corpus callosum	--	--		

Note: **SARS-CoV-2 RNA was isolated and sequenced on both Illumina and PacBio platforms. *SARS-CoV-2 RNA was isolated and only sequenced via Illumina sequencing. L: left, R: right, IV: intravenous, Avg.: average, N: nucleocapsid gene, ng: nanograms, uL: microliters. Empty cells indicate no data was collected. Dashes represent instances where data was collected, but the results were either negative or below the limit of detection (ddPCR, sgRNA) or poor quality after sequencing (variant analyses).

Table S2. Plaque isolation

Sample name	virus isolation modified Vero E6 cells				virus isolation Vero-TMPRSS2 cells				
	undiluted	1:10	Cq	copies/mL	Dilution Plaques observed (1:6)	Plaques inoculated	CPE observed	Harvested	Sequenced
nasal placode	+(2/2)	+(2/2)	22.71	676,800,460.62	1 to 2	8	4	4	3
sinus turbinate	+(2/2)	+(2/2)	20.99	2,119,793,373.85	1	3	1	1	1
(L) inferior lobe	+(2/2)	+(1/2)	21.78	1,253,191,514.12	3 to 4	12	8 to 10	8	3
proximal trachea	+(2/2)	-(0/2)	20.52	2,898,848,228.83	3 to 4	12	8 to 10	8	3
bone rib	-(0/2)	-(0/2)	--	0.00					
(L) bronchus	+(1/2)	-(0/2)	20.63	2,696,930,903.07	3 to 4	12	8 to 10	8	3
(L) eye optic nerve	-(0/2)	-(0/2)	--	0.00					
pericardium	-(0/2)	-(0/2)	--	0.00					
stomach	-(0/2)	-(0/2)	--	0.00					
(R) eye optic nerve	-(0/2)	-(0/2)	--	0.00					
thoracic lymph node	-(0/2)	-(0/2)	--	0.00					
appendix	-(0/2)	-(0/2)	37.76	29,573.61					
(L) kidney	-(0/2)	-(0/2)	--	0.00					
(R) eye cornea	-(0/2)	-(0/2)	--	0.00					
tongue	+(2/2)	+(1/2)	20.97	2,150,283,628.85	3 to 4	12	8 to 10	8	3
parotid salivary gland	-(0/2)	-(0/2)	--	0.00					

Table S3. Relative frequency of infectious virus isolated from different tissue sites.

Tissue site	Plaque #	Isolate ID	S: R19T	S: E406D	S: V445A	S: G446V	S: L455F	S: S477N	S: K478E	S: Q493K	S: T573I	S: G1267R	ORF6: L4F	ORF8: A55V	N: P142Q
(L) bronchus	1	1	1.00		1.00			1.00	1.00	0.93					
	2		1.00		1.00			1.00	1.00	1.00					
	4		1.00		1.00			1.00	0.99	1.00					
(L) inferior lobe	1		1.00		0.96			1.00	1.00	1.00					
	2		1.00		1.00			1.00	1.00	0.88					
	3		1.00		1.00			1.00	1.00	1.00					
proximal trachea	1		1.00		1.00			1.00	1.00	1.00					
	2		1.00		1.00			1.00	1.00	1.00					
	3		1.00		1.00			1.00	1.00	1.00					
sinus turbinate	2	2			1.00			1.00		1.00					
nasal placode	1	3		1.00		1.00	1.00				1.00				
	2	4		1.00	0.99			1.00		1.00				1.00	
	7			1.00	1.00			1.00		1.00				1.00	
tongue	1	5			1.00			1.00		1.00		1.00			1.00
	3				0.64			1.00		1.00		0.60			0.82
	6				1.00			1.00		1.00			0.99		
